# Efficient plasmid transfer via natural competence in a microbial co‐culture

**DOI:** 10.15252/msb.202211406

**Published:** 2023-01-30

**Authors:** Yu‐Yu Cheng, Zhichao Zhou, James M Papadopoulos, Jason D Zuke, Tanya G Falbel, Karthik Anantharaman, Briana M Burton, Ophelia S Venturelli

**Affiliations:** ^1^ Department of Biochemistry University of Wisconsin Madison WI USA; ^2^ Department of Bacteriology University of Wisconsin Madison WI USA; ^3^ Department of Chemical & Biological Engineering University of Wisconsin Madison WI USA

**Keywords:** extracellular DNA, horizontal gene transfer, microbial co‐culture, natural competence, SOS response, Microbiology, Virology & Host Pathogen Interaction

## Abstract

The molecular and ecological factors shaping horizontal gene transfer (HGT) via natural transformation in microbial communities are largely unknown, which is critical for understanding the emergence of antibiotic‐resistant pathogens. We investigate key factors shaping HGT in a microbial co‐culture by quantifying extracellular DNA release, species growth, and HGT efficiency over time. In the co‐culture, plasmid release and HGT efficiency are significantly enhanced than in the respective monocultures. The donor is a key determinant of HGT efficiency as plasmids induce the SOS response, enter a multimerized state, and are released in high concentrations, enabling efficient HGT. However, HGT is reduced in response to high donor lysis rates. HGT is independent of the donor viability state as both live and dead cells transfer the plasmid with high efficiency. In sum, plasmid HGT via natural transformation depends on the interplay of plasmid properties, donor stress responses and lysis rates, and interspecies interactions.

## Graphical abstract

Plasmid multimerization, donor stress response and donor lysis rate are major determinants of horizontal gene transfer via natural competence in a microbial co‐culture.

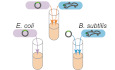

## Introduction

Horizontal gene transfer (HGT) is a major mechanism of genetic variation in microbial communities that enables the acquisition of new functional capabilities (Soucy *et al*, 2015). Horizontally acquired sequences can provide a selective advantage by facilitating evolutionary adaptation to changing environmental conditions (Ropars *et al*, 2015). Conjugation and natural transformation are prevalent processes that enable HGT in bacterial communities. Conjugation involves cell‐to‐cell contact between a donor and recipient cell, and thus, the live donor cell actively participates in the HGT process (Clarke *et al*, 2008). By contrast, extracellular DNA (eDNA) can be acquired by a naturally competent recipient cell in the absence of living donor cells (Overballe‐Petersen *et al*, 2013). The capability for natural competence is widespread across Gram‐positive and Gram‐negative bacteria (Johnston *et al*, 2014), including many bacterial pathogens (Lerminiaux & Cameron, 2019). For example, interspecies or intrastrain gene transfer (Hakenbeck *et al*, 1999; Sauerbier *et al*, 2012; Jensen *et al*, 2015) via natural transformation is implicated in the ability of *Streptococcus pneumoniae* to adapt and persist on a human host.

eDNA derived from bacteria is prevalent in natural environments (Nagler *et al*, 2018). The potential biological functions of eDNA and the mechanisms mediating DNA release vary across bacterial species and environmental contexts (Ibáñez de Aldecoa *et al*, 2017). eDNA can be released through autolysis, active secretion, or cell death and is involved in biofilm formation, DNA repair, nutrient utilization, and gene transfer (Finkel & Kolter, 2001; Whitchurch *et al*, 2002; Prudhomme *et al*, 2006; Zafra *et al*, 2012). Environmental stressors such as bactericidal antibiotics, temperature shifts, chemicals, and enzymes can also modify cell viability and thus the rate of eDNA release (Boman & Eriksson, 1963; Shehadul Islam *et al*, 2017). In addition, interactions between donors and recipients have been shown to influence eDNA release and the frequency of HGT in microbial co‐cultures. For instance, naturally competent species can exploit predation to enhance DNA release from donor cells to achieve efficient DNA acquisition. For example, *Acinetobacter baumannii*, *Vibrio cholerae*, and *S. pneumoniae* can use type‐VI secretion systems or bacteriocins to enhance DNA release via lysis of the donor (Borgeaud *et al*, 2015; Wholey *et al*, 2016; Cooper *et al*, 2017). In addition, the presence of a donor strain has been shown to enhance the frequency of intrastrain gene transfer in *Bacillus subtilis*, *Porphyromonas gingivalis*, and *Pseudomonas stutzeri*, as well as interspecies gene transfer between the donor *Escherichia coli* and recipient *Vibrio* species (Stewart *et al*, 1983; Paul *et al*, 1992; Tribble *et al*, 2012; Zhang *et al*, 2018). However, the mechanisms driving these interactions are largely unknown.

At the molecular level, the DNA strand exchange protein and master regulator of the SOS response RecA has been shown to play an important role in facilitating HGT (Pavlopoulou, 2018). In the recipient, RecA mediates homologous recombination with incoming DNA with sufficient homology to the recipient genome (Kowalczykowski & Eggleston, 1994). In the donor, RecA plays an important role in HGT via transduction and conjugation (Johnson *et al*, 1981; Beaber *et al*, 2004). In response to DNA damage, RecA binds to single‐stranded DNA and inactivates the LexA repressor that regulates SOS response genes (global response to DNA damage; Maslowska *et al*, 2019). Depending on the degree of DNA damage, the SOS response activates the expression of error‐prone DNA polymerases, inhibitors of cell division, and proteins that promote cell death (Simmons *et al*, 2008). The repressors in the phages and the integrative and conjugative elements share homology with LexA, which in turn enhances the rates of transduction and conjugation during DNA damage (Johnson *et al*, 1981; Beaber *et al*, 2004). In microbial communities, the role of RecA in donor species on HGT via natural competence has not been investigated.

To address these gaps, we construct a microbial co‐culture composed of the donor *E. coli* and recipient *B. subtilis* to interrogate the contributions of environmental and molecular factors on eDNA release and efficiency of HGT via natural competence. These two species are evolutionarily distant and thus can provide fundamental insights into the mechanisms of HGT within diverse microbial communities. We demonstrate that eDNA release and HGT efficiency via natural competence depends on the initial culture densities of both donor and recipient, mirroring conjugation (Lopatkin *et al*, 2016). Notably, *recA* in the *E. coli* donor has a major impact on plasmid transfer frequencies by enhancing plasmid multimerization and eDNA release. Using an inducible control of cell lysis, we show that a high rate of donor lysis inhibits plasmid transfer due to the release of both donor genomic DNA (gDNA) and DNases. By comparison, live, heat‐killed, or antibiotic‐inhibited donor cells can efficiently release eDNA and yield high HGT efficiencies in the microbial co‐culture. These results suggest that the donor could efficiently transfer plasmids to naturally competent bacteria in microbial communities with or without perturbations. Bioinformatic analysis identifies *E. coli* plasmid replication origins in the genomes of wild‐type *Bacillus* isolates and other naturally competent bacteria, suggesting that plasmids may transfer via natural transformation. In sum, our results provide key insights into the molecular and environmental factors influencing HGT frequency via natural competence in microbial communities.

## Results

### Interspecies plasmid HGT via natural transformation is efficient in a microbial co‐culture

**Figure 1:**
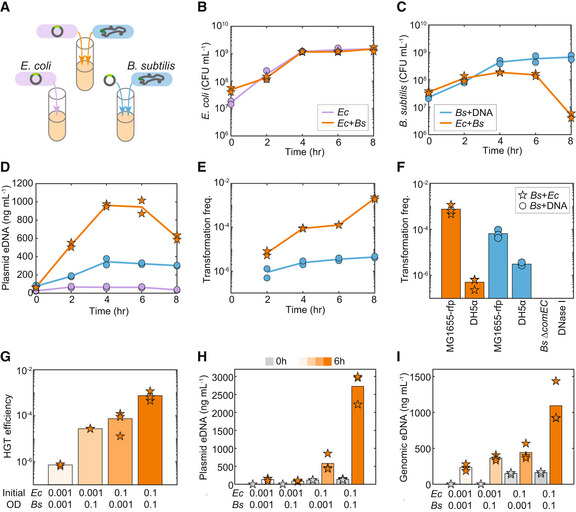
Plasmid horizontal gene transfer via natural transformation is highly efficient in the co‐culture A
Schematic of experimental design to characterize the temporal changes in plasmid horizontal gene transfer, species abundance, and extracellular plasmid release in *Escherichia coli* monoculture (purple), *Bacillus subtilis* monoculture supplemented with 100 ng/ml plasmid DNA (blue) or co‐culture composed of *E. coli* harboring a plasmid and *B. subtilis* (orange). The pBB275 plasmid harbored a resistance gene flanked by two sequences homologous to *B. subtilis* for integration onto the *B. subtilis* genome. *B. subtilis* was engineered to harbor a xylose‐inducible master regulator for competence *comK* for enhancing transformation efficiency in the co‐culture.B–E
Time‐series measurements of (B) *E. coli* abundance, (C) *B. subtilis* abundance, (D) extracellular plasmid concentration, and (E) transformation frequency of the plasmid in monoculture and co‐culture.F
Bar plot of transformation frequency of the pBB275 plasmid at 6 h in the *B. subtilis* monoculture or co‐culture. *E. coli* MG1655‐rfp or *E. coli* DH5α was used as the donor species in the co‐culture. In the monoculture, 1 μg/ml plasmid DNA derived from *E. coli* MG1655‐rfp or DH5α was introduced. The transformation frequency was below the detection limit in the co‐culture composed of *E. coli* M1655‐rfp and *B. subtilis* ∆*comEC* or the co‐culture composed of *E. coli* MG1655‐rfp and *B. subtilis* in the presence of 1 unit/ml DNase I.G
HGT efficiency (i.e., transformation frequency in the co‐culture) of the pBB275 plasmid at 6 h in the co‐cultures composed *E. coli* MG1655‐rfp and *B. subtilis* inoculated at different initial abundances (absorbances at 600 nm or OD_600_).H
Bar plot of measured extracellular pBB275 plasmid concentration at 0 and 6 h in co‐cultures inoculated with different initial culture densities of *E. coli* MG1655‐rfp and *B. subtilis*.I
Extracellular *E. coli* genomic MG1655‐rfp concentration at 0 and 6 h in co‐cultures inoculated with different initial culture densities of *E. coli* MG1655‐rfp and *B. subtilis*. Data information: All time‐series experiments in (B–E) had two biological replicates. All single time measurements in (F–I) had three biological replicates. Lines and bars represent the average of the biological replicates. Source data are available online for this figure.

To investigate HGT mediated by natural competence, we constructed a microbial co‐culture composed of the donor *E. coli* and recipient *B. subtilis* (Fig 1A). This co‐culture enabled quantification of species abundance, eDNA release, and HGT frequency over time. The *E. coli* donor harbored an integrative plasmid pBB275 with a spectinomycin resistance gene (*specR*) flanked by two 500‐bp sequences homologous to *B. subtilis* PY79 locus *ycgO*. Once the plasmid is released from *E. coli* and taken up by *B. subtilis*, it can be integrated onto the *B. subtilis* genome via homologous recombination. The donor *E. coli* strain (MG1655‐rfp) has a constitutively expressed red fluorescent gene (*rfp*) on the genome to enable fluorescent imaging. To enhance transformation efficiency (~100‐fold) in rich media that supports the growth of both species, a xylose‐inducible master regulator for competence (*comK*) was introduced into *B. subtilis* (Zhang & Zhang, 2011).

To understand the dynamics of HGT, we measured the colony‐forming units (CFU/ml) of the *E. coli* donor, *B. subtilis* recipient, and *B. subtilis* transformants over time in monoculture and co‐culture conditions (Fig 1A–C). To investigate the temporal changes in eDNA release, we performed quantitative real‐time PCR (qPCR) measurements of the pBB275 plasmid released from *E. coli* (MG1655‐rfp) in monoculture and co‐culture of *E. coli* (MG1655‐rfp) and *B. subtilis* (Fig 1A and D). To evaluate the efficiency of HGT, we quantified the transformation frequency of the co‐culture or *B. subtilis* monoculture supplemented with purified pBB275 plasmid (100 ng/ml) derived from the cloning strain *E. coli* DH5α (Fig 1A and E). Transformation frequency was defined as the ratio of the number of *B. subtilis* transformants plated on selective media to the total number of *B. subtilis* cells. Based on a titration of plasmid concentration in *B. subtilis* monoculture, 100 ng/ml was close to the saturated regime of transformation efficiency in rich media supplemented with 50 mM xylose (Appendix Fig S1A). This indicates that plasmid concentrations higher than 100 ng/ml would not substantially enhance transformation frequency.

The growth of *E. coli* was similar in the presence and absence of *B. subtilis*, indicating the absence of an interspecies interaction (Fig 1B). However, the growth of *B. subtilis* after 4 h was reduced in the presence of *E. coli*, indicating a negative interspecies interaction from *E. coli* to *B. subtilis* (Fig 1C). The fluorescently labeled *E. coli* and *B. subtilis* aggregated together, suggesting that cell‐to‐cell contact with *E. coli* may influence *B. subtilis* growth in the co‐culture (Appendix Fig S1B). The growth inhibition was observed for both the wild‐type and engineered *B. subtilis* strains (Fig 1C and Appendix Fig S1C). In addition, eDNA released from *E. coli* was substantially enhanced in the presence of *B. subtilis* than in the *E. coli* monoculture (Fig 1D, orange vs. purple line). The externally supplemented plasmid concentration moderately increased over time in the *B. subtilis* monoculture, possibly due to autolysis (i.e., regulated lysis) of *B. subtilis* (Zafra *et al*, 2012; Fig 1D, blue line). The transformation frequencies of both the engineered and wild‐type *B. subtilis* strains were significantly enhanced (~10^2^–10^3^ fold at 6 h) in the co‐culture than in the *B. subtilis* monocultures supplemented with 100 ng/ml plasmid derived from *E. coli* DH5α (Fig 1E and Appendix Fig S1D). For further characterizations, we focused on the transformation frequency at 6 h due to the high *B. subtilis* abundance and high transformation frequency observed in the co‐culture (Fig 1C and E).

We considered if the differences in the genetic background of the *E. coli* donor (i.e., purified plasmid derived from DH5α or released plasmid from MG1655‐rfp) could drive the high transformation frequency observed in the co‐culture. Indeed, multimerized plasmids could be isolated from *E. coli* MG1655‐rfp, consistent with a previous study demonstrating plasmid multimerization in *recA* ^+^ *E. coli* (Bedbrook & Ausubel, 1976; Appendix Fig S1E). *B. subtilis* monoculture supplemented with plasmid derived from MG1655‐rfp displayed ~10‐fold higher transformation frequency than when supplemented with plasmid derived from DH5α (Fig 1F, blue bars, Appendix Fig S1A). Notably, the transformation frequency in the co‐culture with *E. coli* DH5α was ~10^3^ lower than in the co‐culture with *E. coli* MG1655‐rfp (Fig 1F, orange bars). This implies that while plasmid multimerization was a key factor that enhanced HGT efficiency (~10‐fold), it did not fully explain the large increase in HGT efficiency observed in the co‐culture with MG1655‐rfp compared with DH5α. The knockout of DNA transport protein gene *comEC* in engineered *B. subtilis* or the addition of DNase I abolished plasmid transfer in the co‐culture with *E. coli* MG1655‐rfp, indicating that the efficient plasmid DNA transfer in the co‐culture was mediated by natural competence (Fig 1F, right two bars).

To investigate how initial cell density could affect HGT efficiency in the co‐culture, we varied the initial cell densities of *E. coli* and *B. subtilis* based on absorbance at 600 nm (OD_600_) from 0.001 to 0.1 and measured species abundance, eDNA release, and HGT efficiency at 6 h. The plasmid eDNA and HGT efficiency increased with the initial cell densities of *E. coli* and *B. subtilis* (Fig 1G and H). The extracellular chromosomal DNA of *E. coli* was significantly higher for co‐cultures with high initial abundance of both species (Fig 1I), suggesting that *E. coli* was more susceptible to cell death in cultures with higher initial cell density. Lower initial *E. coli* abundance resulted in higher abundance of *B. subtilis* at 6 h, consistent with *E. coli'*s negative impact on the growth of *B. subtilis* (Appendix Fig S1F). In sum, initial species abundance was a critical variable shaping HGT efficiency and eDNA release in the co‐culture.

We investigated whether chromosomal DNA could transfer from *E. coli* to *B. subtilis* in the co‐culture via natural competence. We constructed an *E. coli* donor with an erythromycin resistance gene flanked by 600‐bp sequences homologous to *B. subtilis* PY79 *yvbJ* on the *E. coli* MG1655 genome (Appendix Fig S2A). Transformants were not observed over 8 h in the co‐culture of the *E. coli* donor strain and engineered *B. subtilis* (detection limit was ~10^−9^; Appendix Fig S2B). By contrast, the *B. subtilis* monoculture supplemented with *E. coli* gDNA (100 ng/ml) displayed low transformation frequency (Appendix Fig S2B). While eDNA release of the *E. coli* donor strain was moderately higher in co‐culture than in monoculture (Appendix Fig S2C, orange vs. purple line), this concentration was not sufficiently high to enable efficient HGT (Appendix Fig S2C, blue line). In this co‐culture, the growth of *B. subtilis* was also inhibited by *E. coli* (Appendix Fig S2D and E). In sum, our results indicate that HGT of plasmid DNA was substantially more efficient than chromosomal DNA via natural competence in the co‐culture.

### Investigating the role of donor *recA* on HGT of plasmid via natural competence

**Figure 2:**
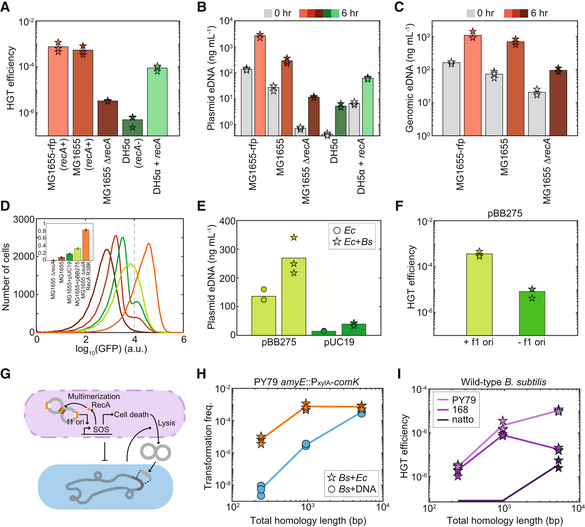
Presence of *recA* in the *Escherichia coli* donor enhances HGT efficiency in the co‐culture A
Bar plot of HGT efficiencies of the pBB275 plasmid in the co‐culture composed of an *E. coli recA* ^+^ or *recA* ^−^ donor and engineered *B. subtilis* at 6 h.B, C
(B) Extracellular pBB275 plasmid concentration or (C) extracellular *E. coli* genomic DNA concentration at 0 or 6 h in the co‐cultures composed of a *recA* ^+^ or *recA* ^−^ *E. coli* plasmid donor and engineered *B. subtilis*.D
Histogram of GFP expression driven by an SOS response promoter in *E. coli* MG1655 ∆*recA*, *E. coli* MG1655, *E. coli* MG1655 harboring pUC19, *E. coli* MG1655 harboring pBB275 or *E. coli* MG1655 ∆*sulA* RecA(E38K) in monoculture measured by flow cytometry. Inset: bar plot of the fraction of *E. coli* expressing high GFP (> 10,000 a.u.) for each strain.E
Extracellular pBB275 or pUC19 plasmid concentration in the *E. coli* MG1655 monoculture or co‐culture with engineered *B. subtilis*.F
HGT efficiencies of pBB275 plasmid (with or without an f1 ori) at 6 h in the co‐culture composed of *E. coli* MG1655 and engineered *B. subtilis*.G
Schematic of the proposed model for the efficient plasmid transfer via natural competence in the co‐culture composed of a *recA* ^+^ *E. coli* donor and recipient *B. subtilis*. The plasmid leads to the activation of the SOS response, which in turn enhances eDNA release and plasmid multimerization via RecA. *B. subtilis* enhances *E. coli* eDNA release even being inhibited by *E. coli*.H
Relationship between total homology length and transformation frequency of pBB275 plasmid in the co‐culture composed of *E. coli* MG1655‐rfp and engineered *B. subtilis* or *B. subtilis* monoculture at 6 h. In the *B. subtilis* monoculture, 1 μg/ml pBB275 plasmid DNA derived from *E. coli* DH5α was introduced.I
Relationship between total homology length and HGT efficiency of the pBB275 plasmid in the co‐culture composed of the DAP‐auxotrophic *E. coli* BW29427 and wild‐type *B. subtilis* PY79, 168 or natto IFO3335 at 6 h. HGT efficiency of pBB275 plasmid with total ~200‐ and ~1,000‐bp homology for *B. subtilis* natto was below the detection limit. Data information: All experiments had three biological replicates. Bars and lines are the average of the biological replicates. Source data are available online for this figure.

We investigated factors influencing the ~10^3^‐fold increase in HGT efficiency in the co‐culture with *E. coli* MG1655‐rfp compared with DH5α (Fig 1F, orange bars). *E. coli* MG1655 and DH5α contain many genetic differences including *recA*. We compared the HGT efficiency of *recA* ^+^ *E. coli* donors (MG1655‐rfp, MG1655, or DH5α + *recA*) with *recA* ^−^ *E. coli* donors (MG1655 ∆*recA* or DH5α) in the co‐culture with engineered *B. subtilis* (Fig 2A). Our results showed that *recA* ^+^ donors displayed substantially higher HGT efficiency in the co‐culture than *recA* ^−^ donors (Fig 2A). Notably, the introduction of a constitutively expressed *recA* in *E. coli* DH5α yielded a ~100‐fold increase in HGT efficiency compared with *E. coli* DH5α, indicating that *recA* is a major determinant of HGT efficiency. Plasmid and chromosomal eDNA were significantly higher in co‐cultures with *E. coli recA* ^+^ donors than *recA* ^−^ donors, indicating that RecA contributes to eDNA release from *E. coli* and enhances HGT efficiency (Fig 2B and C). However, the eDNA increase did not trend with *E. coli* abundance at 6 h in the co‐culture or *E. coli* growth rate in monoculture (Appendix Fig S3). These strains displayed similar doubling times in monoculture, with MG1655‐rfp and DH5α + *recA* displaying the fastest and slowest growth rates, respectively (Appendix Fig S3C). These results suggest that RecA may facilitate plasmid transfer by increasing the death rate of a subpopulation of donor cells while not substantially altering growth.

Since RecA has been shown to induce cell death via the SOS response (Erental *et al*, 2014), we tested whether the integrative plasmid pBB275 induced the SOS response of *recA* ^+^ *E. coli*, which in turn could enhance eDNA release. To quantify the activity of the SOS response, we constructed an SOS response reporter P_*sulA*_‐*gfp* on a different plasmid (McCool *et al*, 2004). To test the reporter, we first introduced the SOS response reporter plasmid into *E. coli* MG1655 ∆*recA*, MG1655, and MG1655 ∆*sulA* RecA(E38K) (constitutively active SOS response (Robinson *et al*, 2015)) without the integrative plasmid. We used flow cytometry to quantify the fluorescence distribution of the SOS response reporter across the *E. coli* population in monoculture. By setting a GFP threshold of 10^4^ a.u. (GFP ON subpopulation), 1% of *E. coli* MG1655 ∆*recA*, 9% of *E. coli* MG1655, and 83% of wild‐type *E. coli* MG1655 ∆*sulA* RecA(E38K) were GFP ON. This implies that the SOS fluorescent reporter can quantify the degree of SOS response in *E. coli* (Fig 2D).

Plasmid pBB275 consists of heterologous DNA sequences including sequences homologous to *B. subtilis* and a phage‐derived replication origin (f1 ori), which could activate the SOS response in *E. coli* (Arís *et al*, 1998; Lee *et al*, 2002; Johnson *et al*, 2019; Appendix Fig S4A). To evaluate the effect of the pBB275 plasmid on the activity of the SOS response, we characterized the SOS response in *E. coli* MG1655 harboring pBB275 and in *E. coli* MG1655 with a plasmid pUC19 that lacks the sequences homologous to *ycgO* in *B. subtilis* and the f1 ori (Appendix Fig S4B). *E. coli* MG1655 harboring pUC19 or pBB275 displayed larger GFP ON subpopulations (18 and 33% of the populations, respectively) than MG1655 lacking these plasmids (9% of the population). In addition, the GFP ON subpopulation in *E. coli* MG1655 harboring pBB275 displayed elongated cell morphologies by microscopy and higher Forward Scatter measured by flow cytometry compared with the GFP ON subpopulation of *E. coli* MG1655 harboring pUC19 (Appendix Fig S5A–D). This elongated cell morphology is consistent with SOS response induction in *E. coli* (Witkin, 1967). In sum, these results show that different plasmids can induce the SOS response in *E. coli* to varying degrees.

Plasmid eDNA release was substantially higher for *E. coli* MG1655 harboring pBB275 than *E. coli* MG1655 harboring pUC19 in monoculture and in the co‐culture with engineered *B. subtilis*. This is consistent with the observed larger fraction of the GFP ON cells for SOS reporter in *E. coli* with pBB275 than pUC19 (Fig 2D and E). The plasmid conformations of pUC19 derived from *recA* ^+^ *E. coli* or *recA* ^−^ *E. coli* were similar (Appendix Fig S5E). This implies that sequences that differentiate pUC19 and pBB275 (e.g., f1 ori and *ycgO*) could contribute to plasmid multimerization. To determine whether the phage replication origin contributed to the high HGT efficiency, we constructed a derivative of pBB275 that lacks the f1 ori (Appendix Fig S4C). The HGT efficiency of pBB275 was substantially higher than pBB275 lacking the f1 ori, indicating that the f1 ori was a critical determinant of HGT efficiency (Fig 2F). The f1 ori has been previously shown to activate the SOS response (Johnson *et al*, 2019). Taken together, this suggests that the f1 ori, SOS response, and plasmid multimerization interact to enhance HGT efficiency in the co‐culture (Fig 2G).

To investigate how the sequence length of *B. subtilis* homologous DNA on the integrative pBB275 plasmid could impact HGT efficiency, we constructed plasmids with homology length 100 or 2,500 bp that also harbored the f1 ori. Plasmids with different homology lengths all formed multimers when derived from *E. coli* MG1655‐rfp and not when derived from *E. coli* DH5α (Appendix Fig S5F). The HGT efficiency of these plasmids was substantially higher in co‐culture than in monoculture supplemented with high concentration of plasmid DNA derived from DH5α (1 μg/ml; Fig 2H). The magnitude of this enhancement in HGT efficiency was maximized (~1,000‐fold) for the plasmid with the shortest homology length (Fig 2H). The plasmid eDNA concentration displayed a nonmonotonic relationship with homology length and was highest for the plasmid with 500‐bp homology (Appendix Fig S5G).

Based on the high HGT efficiency of these plasmids, we tested whether wild‐type *B. subtilis* can be directly transformed in the co‐culture in a rich media that does not typically induce natural competence activity. To this end, we transformed individual plasmids into a diaminopimelic acid (DAP) auxotrophic *E. coli* strain. We co‐cultured the individual auxotrophic *E. coli* donors with wild‐type *B. subtilis* PY79, 168, or natto IFO3335 as recipients (Goto & Kunioka, 1992; Zeigler *et al*, 2008; Wang *et al*, 2015). By selecting for spectinomycin‐resistant cells in the absence of DAP, HGT occurred in co‐culture for all three *B. subtilis* strains with varying transformation efficiencies (Fig 2I). In sum, our results demonstrate that the plasmids can transfer efficiently from *recA* ^+^ *E. coli* donor strains to different *B. subtilis* recipient strains via natural competence.

### High donor lysis rate reduces HGT efficiency

**Figure 3:**
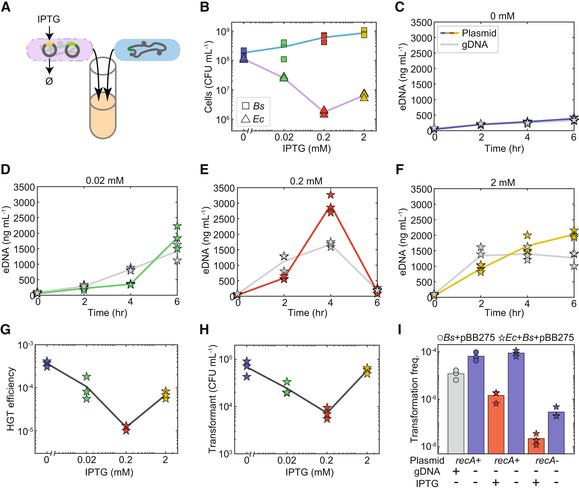
High *Escherichia coli* donor cell lysis rates inhibit HGT efficiency in the co‐culture A
Schematic of the co‐culture experiment composed of engineered *B. subtilis* and *E. coli* MG1655‐rfp harboring pBB275 integrative plasmid and an IPTG‐inducible lysis gene on a second plasmid.B
*E. coli* and *B. subtilis* abundances in the co‐culture in the presence of 0, 0.02, 0.2 or 2 mM IPTG at 6 h.C–F
Time‐series measurements of extracellular plasmid or *E. coli* gDNA concentrations in the co‐culture in the presence of (C) 0 mM, (D) 0.02 mM, (E) 0.2 mM or (F) 2 mM IPTG.G
HGT efficiency of the pBB275 plasmid in the co‐culture in the presence of 0, 0.02, 0.2 or 2 mM IPTG at 6 h.H
Abundance of transformants in the co‐culture in the presence of 0, 0.02, 0.2 or 2 mM IPTG at 6 h.I
Bar plot of transformation frequencies of engineered *B. subtilis* in monoculture or co‐culture with *E. coli* harboring an IPTG‐inducible lysis gene. In the *B. subtilis* monoculture or co‐culture, 1 μg/ml pBB275 plasmid DNA derived from *E. coli* MG1655‐rfp (*recA* ^+^) or DH5α (*recA* ^−^) was externally introduced. The presence of 1 μg/ml genomic DNA derived from *E. coli* MG1655‐rfp in *B. subtilis* monoculture or 0.2 mM IPTG in co‐culture reduced the transformation frequency of the pBB275 plasmid DNA at 6 h. Data information: Each experiment had three biological replicates. Lines and bars are the average of the biological replicates. Source data are available online for this figure.

Bacteria are continuously confronted with biotic and abiotic environmental stimuli that impact cellular growth rates and viability (Maurice *et al*, 2013; García‐Bayona & Comstock, 2018). We investigated how different perturbations on donor growth could impact HGT efficiency in our microbial co‐culture. To precisely control the lysis rate of the donor, we introduced an Isopropyl β‐D‐1‐thiogalactopyranoside (IPTG)‐inducible phage φX174 lysis gene *E* into the *E. coli* MG1655‐rfp donor strain on a second plasmid in addition to the integrative plasmid pBB275 (Fig 3A). With the addition of IPTG, *E. coli* abundance decreased at 6 h, consistent with induced lysis (Fig 3B). In addition, *B. subtilis* grew to a moderately higher abundance, consistent with a weakened negative interaction from *E. coli* to *B. subtilis* (Fig 3B). The concentrations of plasmid and chromosomal eDNA of *E. coli* were substantially higher in the presence of IPTG, consistent with the high lysis rate and reduced abundance of *E. coli* (Fig 3B–F). Notably, despite the high eDNA concentrations in the presence of IPTG, the HGT efficiency and abundance of transformants decreased over a range of IPTG concentrations (Fig 3G and H).

To test whether the released *E. coli* gDNA could compete with plasmid for transformation, we supplemented a high concentration of *E. coli* MG1655‐rfp gDNA (1 μg/ml) into the *B. subtilis* monoculture that also contained a high concentration of pBB275 plasmid DNA (1 μg/ml). The presence of *E. coli* gDNA inhibited plasmid transformation of the *B. subtilis* monoculture (Fig 3I, first two bars). This implies that gDNA could compete with plasmid for DNA uptake and/or limit homologous recombination efficiency. To test whether the *E. coli* lysate contained an additional factor that inhibits plasmid HGT in addition to gDNA, we co‐cultured *B. subtilis* with an *E. coli* MG1655‐rfp strain harboring only the plasmid containing IPTG‐inducible lysis gene. To control the plasmid concentration, we externally supplemented a high concentration of pBB275 (1 μg/ml) derived from DH5α (monomeric) or MG1655‐rfp (multimeric) into separate cultures. The transformation frequencies of externally supplemented plasmids were substantially reduced in the presence of IPTG (0.2 mM) and were lower than the *B. subtilis* monoculture supplemented with gDNA. This indicates that both gDNA competition and donor cell lysis reduced the transformation frequency of both monomeric and multimeric plasmids (Fig 3I, last four bars). The externally supplemented pBB275 plasmid was degraded in the supernatants of the co‐cultures in the presence of IPTG at 6 h (Appendix Fig S6). This implies that DNases released from lysed *E. coli* could also inhibit plasmid HGT in addition to released gDNA. These data indicate that maximizing donor cell lysis did not achieve a corresponding increase in HGT efficiency. In sum, *E. coli* harboring a plasmid that enhanced the activity of the SOS response displayed the highest HGT efficiency, suggesting that donor cell lysis rates can be optimized to maximize HGT efficiency.

### Live or dead donor cells contribute to efficient plasmid transfer

**Figure 4:**
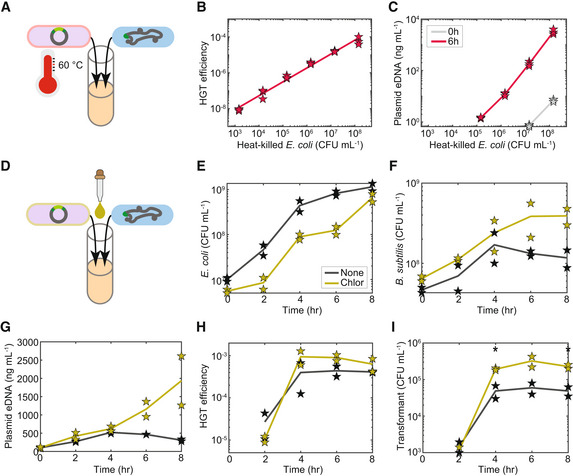
High HGT efficiency in the microbial co‐culture is independent of *Escherichia coli* donor viability state A
Schematic of the experimental design to determine the impact of donor species viability on HGT efficiency and plasmid DNA release. The microbial co‐culture was composed of engineered *B. subtilis* and heat‐killed *E. coli* MG1655‐rfp harboring pBB275.B
Scatter plot of the initial abundance of heat‐killed *E. coli* MG1655‐rfp and HGT efficiency in the co‐culture at 6 h. Line represents data fit to the linear equation *y* = 0.74*x − 10.37 with the coefficient of determination *R* ^2^ = 0.9839 where *x* and *y* are the log10 transformed abundance of heat‐killed *E. coli* density and log10 transformed HGT efficiency, respectively.C
Scatter plot of the initial abundance of heat‐killed *E. coli* MG1655‐rfp and extracellular pBB275 plasmid concentration at 0 or 6 h in the co‐culture.D
Schematic of the experimental design to determine the impact of chloramphenicol on HGT efficiency and plasmid DNA release in the co‐culture composed of engineered *B. subtilis* and *E. coli* MG1655‐rfp. *B. subtilis* harbored a chloramphenicol resistance gene.E–I
Time‐series measurements of (E) the abundance of *E. coli* MG1655‐rfp, (F) the abundance of engineered *B. subtilis*, (G) extracellular pBB275 plasmid concentration, (H) HGT efficiency of the pBB275 plasmid, and (I) abundance of transformants in the co‐culture in the presence or absence of chloramphenicol (5 μg/ml). Unpaired *t*‐test was used to determine whether the abundances in the presence and absence of chloramphenicol in (I) were statistically different. Stars (*) indicate *P*‐value of 0.0237 and 0.0283 for 4 and 8 h, respectively. Data information: Single time point measurements in (B and C) had three biological replicates. Time‐series measurements in (E–I) had two biological replicates. Lines denote the average of the biological replicates. Source data are available online for this figure.

To determine whether live and metabolically active donor cells are contributing to the observed efficiency of HGT in the co‐culture, the *E. coli* donor was killed by incubating at 60°C for 30 min (Usajewicz & Nalepa, 2006). This heat treatment did not induce substantial eDNA release when compared to eDNA release of live cells (Appendix Fig S7A and B). The log‐transformed HGT efficiency of heat‐killed *E. coli* co‐cultured with *B. subtilis* was linearly related to the log transform of the initial cell density of heat‐treated *E. coli* (Fig 4A and B). In addition, the plasmid eDNA concentration at 6 h was linearly related to the initial cell density of heat‐killed *E. coli* (Fig 4C). This indicates that plasmids can be efficiently released from dead *E. coli* cells and transferred to *B. subtilis* in the co‐culture.

To further investigate this question, we supplemented a sublethal concentration of the bacteriostatic antibiotic chloramphenicol (5 μg/ml) into the co‐culture that selectively inhibits *E. coli* growth by blocking protein synthesis of *E. coli* (Fig 4D). The engineered *B. subtilis* harbored a chloramphenicol resistance gene and was not impacted by the presence of the antibiotic. In the presence of chloramphenicol, the growth of *E. coli* was reduced, whereas *B. subtilis* abundance was enhanced at later times, consistent with a diminished negative interspecies interaction impacting *B. subtilis* (Fig 4E and F). In addition, the eDNA release of plasmid and chromosome was enhanced at later times in the presence of chloramphenicol (Frenkel & Bremer, 1986; Fig 4G and Appendix Fig S7C). The HGT efficiency was similar with or without chloramphenicol, indicating that it was not affected by the growth rate of *E. coli* donor (Fig 4E and H). However, the number of *B. subtilis* transformants were higher in the co‐culture due to the higher abundance of *B. subtilis* in the presence of the antibiotic (Fig 4F and I). In sum, donor cells, either live or dead, can release eDNA and yield efficient HGT in microbial co‐cultures.

### Plasmid replication origins in wild‐type *Bacillus* and non‐*Bacillus* genomes

**Figure 5:**
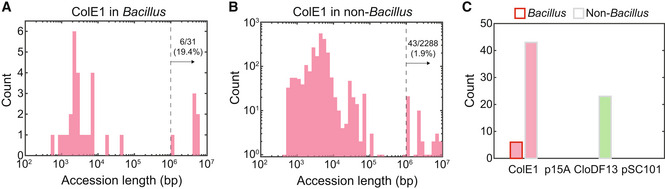
Genomic sequence analysis identifies *Escherichia coli* plasmid replication origins in bacterial genomes A
Histogram of the sequence lengths of 31 *Bacillus* containing a ColE1 replication origin.B
Histogram of the sequence lengths of 2,288 strains excluding members of *E. coli* and *Bacillus* containing a ColE1 replication origin.C
Histogram of the number of *E. coli* plasmid replication origins ColE1, p15A, CloDF13 or pSC101 identified in *Bacillus* or non‐*Bacillus* DNA sequences with a sequence length larger than 10^6^ bp (indicating potential genomic integration). ColE1 was found in six *Bacillus* genomes and 43 non‐*Bacillus* genomes. CloDF13 was found in 23 non‐*Bacillus* genomes. p15A and pSC101 were not detected in this NCBI RefSeq dataset.

Many constructed *E. coli* plasmids contain sequences that could enhance their release into the environment via mechanisms such as the activation of the SOS response. Therefore, we tested whether *E. coli* plasmids were present in other bacteria beyond our tested strains using bioinformatics. We searched for the *E. coli* plasmid replication origin ColE1 sequence (present in pBB275) in the NCBI Reference Sequence Database (RefSeq) by nucleotide BLAST. We found that 31 *Bacillus* strains from a total of 77 *Bacillus* hits harbored ColE1 with high coverage and similarity (> 80% coverage and > 90% identity; Appendix Fig S8A and Dataset EV1). Further analysis of the sequence length greater than threshold (10^6^ bp) revealed the presence of ColE1 in six *Bacillus* genomes, including *B. subtilis* laboratory strains and three wild‐type *Bacillus* isolates (Fig 5A and Appendix Fig S9). Notably, other plasmid replication origins including p15A and CloDF13 did not return as many hits with high percent identity and percent coverage and no hits were observed for pSC101 (Appendix Fig S8B and C, and Dataset EV1).

A similar analysis for non‐*Bacillus* strains revealed that ColE1 and CloDF13 are present in the genomes of 43 and 23 diverse bacteria, respectively (Fig 5B and C, and Appendix Fig S8D–J and Dataset EV1). The replication origin ColE1 was found in diverse wild‐type environmental isolates and laboratory strains, including *Neisseria meningitidis*, which is known to be naturally competent (Berry *et al*, 2013; Appendix Fig S9A). By contrast, CloDF13 was found mostly in *Klebsiella pneumoniae* (Dataset EV1), which may have occurred via conjugation or transduction (Haudiquet *et al*, 2021). Sequence analysis revealed that ColE1 was associated with antibiotic resistance genes and heterologous genes in the genomes of five *Bacillus* strains but not associated with conjugation genes (Appendix Fig S9B). In sum, the presence of plasmid replication origins in the genomes of naturally competent bacteria suggests that *E. coli* plasmids may have transferred to other bacteria via natural transformation.

## Discussion

We used a microbial co‐culture to characterize the molecular and environmental factors shaping HGT via natural competence. Our results demonstrate that plasmid transfer via natural competence can be highly efficient in microbial co‐cultures. We identified several key mechanisms that enable efficient plasmid HGT. Specifically, we show that higher donor eDNA release in the microbial co‐culture and plasmid multimerization enhanced HGT. The eDNA release and natural transformation efficiency also depend on the initial densities of donor and recipient in the co‐culture. The SOS response in the donor *E. coli* species plays a major role in triggering eDNA release, which in turn enhances the efficiency of HGT in the co‐culture. Efficient HGT can be achieved with live and dead donor cells, which demonstrates that purified DNA or cell lysate is not necessary for achieving successful transformation (Kaneko & Itaya, 2010; Juhas *et al*, 2016). These molecular and ecological mechanisms enabling efficient HGT via natural competence could also impact the efficiency of HGT in multispecies communities and natural environments. For example, plasmids isolated from *Staphylococcus aureus* have been shown to be multimerized and transformable, which may lead to the emergence of multidrug‐resistant strains via natural transformation (Canosi *et al*, 1978; Maree *et al*, 2022).

RecA has been shown to be critical for plasmid multimerization in *E. coli* (Bedbrook & Ausubel, 1976) and has only been implicated in cell death via activation of the SOS response in monoculture (Erental *et al*, 2014). To our knowledge, the role of RecA in enhancing eDNA release and HGT efficiency in microbial communities has not been explored. Future studies can examine how different plasmid features could induce strong SOS response and plasmid multimerization (Arís *et al*, 1998; Lee *et al*, 2002; Johnson *et al*, 2019). For example, it is unknown whether the phage replication origin induces strong SOS response via forming single‐stranded DNA and thus triggers the SOS response (Johnson *et al*, 2019). A recent study showed that replication–transcription conflicts on plasmids can lead to plasmid multimerization (Wein *et al*, 2019). In addition, future work will investigate how the degree of population heterogeneity in the donor strain SOS response or the recipient natural competence activity quantitatively shapes HGT efficiency. Future studies using time‐lapse fluorescence microscopy could allow deeper understanding of the interspecies HGT process at the single‐cell level (Cooper *et al*, 2017). Bioinformatic analysis suggests that *E. coli* plasmids can transfer to diverse bacteria including naturally competent bacteria. These results suggest that biocontainment of engineered plasmids is crucial to limit their dissemination to other bacteria in the environment (Arnolds *et al*, 2021).

## Materials and Methods

### Plasmids and bacterial strains

To construct the integrative plasmid pBB275, two ~500‐bp *B. subtilis* PY79 *ycgO* sequences were cloned upstream and downstream of a spectinomycin resistance gene (*specR*) on a ColE1 plasmid. Two other pBB275 plasmids with ~100‐ and ~2,500‐bp *ycgO* homology were also constructed. pUC19 plasmid was purchased from New England Biolabs. The integration region (two *ycgO* sequences and *specR*) on the pBB275 plasmid was cloned onto pUC19 to replace the *lacZα* gene for the construction of plasmid pBB275 without f1 ori. To introduce a constitutively expressed RecA into *E. coli* DH5α, the *recA* gene was PCR amplified from *E. coli* MG1655 gDNA and cloned onto a p15A plasmid (pBbA6k_J23100_recA), where RecA is expressed from P_J23100_ promoter. The SOS response reporter plasmid pBbA6c_PsulA_sfGFP was constructed by introducing 69‐bp P_sulA_ promoter sequence to the upstream of green fluorescent protein gene super‐folder *gfp* (*sfgfp*). The lysis plasmid pYC01 was modified from plasmid pCSaE500, a gift from Lingchong You (Addgene Plasmid #53182). The P_ampC_ promoter of the phage φX174 lysis gene *E* on the pCSaE500 plasmid was replaced with the P_A1lacO‐1_ promoter (Lutz & Bujard, 1997). Plasmids used in this study are listed in Appendix Table S1.

To construct the *E. coli* MG1655‐rfp, a constitutively expressed P_J23100_‐*rfp* was integrated into the *E. coli* MG1655 *caiE* locus using the CRISPR gene editing technique (Egbert *et al*, 2019). To construct the *E. coli* gDNA donor, an erythromycin resistance gene (*ermR*) flanked by two ~600‐bp *B. subtilis* PY79 *yvbJ* sequences was integrated into the *E. coli* MG1655 *caiE* locus using the same CRISPR method. *E. coli* MG1655 ∆*recA* and *E. coli* MG1655 ∆*sulA* RecA(E38K) are gifts from Dr. Michael Cox. To transform wild‐type *B. subtilis* directly in the co‐culture, the DAP‐auxotrophic *E. coli* BW29427 (*E. coli* Genetic Stock Center, CGSC) was used as the plasmid donor. Growth of *E. coli* BW29427 required 25 μM 2,6‐Diaminopimelic acid (DAP, MilliporeSigma) supplemented in LB. *B. subtilis* PY79 was introduced a xylose‐inducible *comK* to increase its transformation efficiency in LB medium (Zhang & Zhang, 2011). To image *B. subtilis* on a fluorescence microscope, a constitutively expressed P_hyperspank_‐*gfp*(Sp) was integrated into the *ycgO* locus (Overkamp *et al*, 2013). Additional antibiotic resistance genes *ermR* and *kanR* were introduced into *B. subtilis* PY79 for selection and gDNA transformation, respectively. *B. subtilis* PY79 strains were constructed using the MC medium for transformation (Konkol *et al*, 2013). MC medium is composed of 10.7 g/l potassium phosphate dibasic (Chem‐Impex International), 5.2 g/l potassium phosphate monobasic (MilliporeSigma), 20 g/l glucose (MilliporeSigma), 0.88 g/l sodium citrate dihydrate (MilliporeSigma), 0.022 g/l ferric ammonium citrate (MilliporeSigma), 1 g/l Oxoid casein hydrolysate (Thermo Fisher Scientific), 2.2 g/l potassium L‐glutamate (MilliporeSigma), and 20 mM magnesium sulfate (MilliporeSigma). A double crossover of the plasmid into *B. subtilis* PY79 genome was confirmed by the replacement of a different antibiotic resistance gene at the integration locus. gDNA of modified *B. subtilis* was then extracted and transformed into a differently modified *B. subtilis* to introduce multiple modifications onto the genome. Bacterial strains used in this study can be found in Appendix Table S2.

### HGT experiments

To start a monoculture or co‐culture of bacteria for transformation, bacteria were first inoculated from the −80°C glycerol stock into 4 ml Lennox LB medium (MilliporeSigma) with selective antibiotics and cultured at 37°C with shaking (250 rpm) for 12 h. OD_600_ of the overnight culture was measured by NanoDrop One (Thermo Fisher Scientific), and the cell culture was diluted to OD_600_ 0.1 in 5 ml LB in a 14 ml Falcon™ Round‐Bottom Tube (Thermo Fisher Scientific). LB was supplemented with 50 mM xylose (Thermo Fisher Scientific) to induce ComK expression in engineered *B. subtilis*. Purified DNA was supplemented into *B. subtilis* monoculture for transformation. Cells in the co‐culture or monoculture were mixed by pipetting and then cultured at 37°C with shaking (250 rpm). Plasmids were extracted using Plasmid Miniprep Kit (Qiagen). gDNA was extracted using DNeasy Blood & Tissue Kit (Qiagen). Cell culture was collected at specific times for qPCR and CFU quantification. To quantify eDNA, cell culture was spun down at 5,000 *g* for 5 min by a table centrifuge (Thermo Fisher Scientific). The supernatant was transferred to a new tube and filtered by 0.2 μm Whatman Puradisc Polyethersulfone Syringe Filter (GE Healthcare), stored at −20°C, and later quantified by qPCR. To count the CFU of the donor or recipient, cell culture was serially diluted in phosphate‐buffered saline (PBS; MilliporeSigma) and plated on selective LB agar plates with specific antibiotics. To count CFU of transformed *B. subtilis*, cells were plated directly on LB agar plates with antibiotics for selection without dilution. LB agar plates were incubated at 37°C, and colonies were counted the next day. Transformation frequency was defined as the ratio of transformed *B. subtilis* to the total *B. subtilis* cells with detection limit ~10^−9^. HGT efficiency was defined as the transformation frequency in the co‐culture with the *E. coli* donor.

For DNase treatment, 1 unit/ml DNase I (Thermo Fisher Scientific) was added to the cell culture. One unit of DNase I completely degrades 1 μg of plasmid DNA in 10 min at 37°C according to the manufacturer's specification. To induce the lysis of *E. coli* in the co‐culture, 0, 0.02, 0.2, or 2 mM IPTG was added at the beginning of the experiment into the co‐culture to induce the lysis gene *E* on pYC01 plasmid. To heat‐kill *E. coli*, overnight *E. coli* cell culture was diluted to OD_600_ 0.1 in LB and cultured at 37°C with shaking (250 rpm) for 6 h. Then, the cells were incubated at 60°C with shaking (250 rpm) for 30 min. Heat‐killed cells were spun down at 5,000 *g* for 5 min. The pellet was resuspended with fresh LB and stored at 4°C for use in the HGT experiment on the next day. CFU counting was performed before and after heat‐killing to confirm that *E. coli* was completely killed. To inhibit *E. coli* growth in the co‐culture, 5 μg/ml chloramphenicol was added at the beginning of the experiment into the co‐culture. *B. subtilis* was not affected by chloramphenicol because of the introduced chloramphenicol acetyltransferase *cat* gene. Antibiotics used in this study were spectinomycin (spec) from Dot Scientific, chloramphenicol (chlor) from MilliporeSigma, MLS (1 μg/ml erythromycin from MilliporeSigma and 25 μg/ml lincomycin from Thermo Fisher Scientific), carbenicillin (carb) from MilliporeSigma, streptomycin (strep) from MilliporeSigma, and kanamycin (kan) from MilliporeSigma.

### qPCR measurement of extracellular DNA concentration

To measure eDNA concentration, filtered supernatant from HGT experiments was added to a qPCR reaction mix containing 2X SsoAdvanced™ Universal Probes Supermix (Bio‐Rad) and PrimeTime qPCR Assays (Integrated DNA Technologies) with 500 nM of forward and reverse primers and 250 nM of probe. For each biological replicate, three technical replicates were performed on the 96‐well PCR plate (Thermo Fisher Scientific). Designed qPCR primers can amplify *specR* on pBB275 plasmid, *ampR* on pUC19 plasmid, *ermR* introduced into *E. coli* MG1655 gDNA donor genome, and *caiE* in the *E. coli* MG1655 plasmid donor genome. qPCR primer sequences are listed in Appendix Table S3. DNA standards of plasmid or gDNA were included in each qPCR run to estimate the eDNA concentration in the collected supernatant. DNA standards were quantified using Quant‐iT dsDNA Assay Kit (Thermo Fisher Scientific). qPCR reactions were performed on the CFX Connect Real‐Time PCR Detection System (Bio‐Rad). eDNA concentration was calculated from the calibration curve of DNA standards.

### Agarose gel electrophoresis for imaging plasmid conformation

To determine the size of plasmids, a 0.8% agarose (MIDSCI) gel was run at 120 V for ~40 min on an electrophoresis equipment (Bio‐Rad). Before electrophoresis, extracted plasmids were mixed with the Purple Gel Loading Dye (6×, no SDS; New England Biolabs) and loaded into wells in the agarose gel. Quick‐Load® Purple 1 kb DNA Ladder (New England Biolabs) or Quick‐Load® 1 kb Extend DNA Ladder (New England Biolabs) was used for the reference of DNA length. Gel images were inverted by ImageJ for visualization purpose.

### Plate reader measurement of bacterial growth

To measure the growth rate of *recA* ^+^ and *recA* ^−^ *E. coli* donors, overnight culture was diluted to OD_600_ 0.1 in LB and transferred to a 96‐well black and clear‐bottom CELLSTAR® format sterile cell culture microplate (Greiner Bio‐One). The plate was sealed with Breathe‐Easy Adhesive Microplate Seal (Thermo Fisher Scientific) and cultured with shaking in the TECAN Spark 10 M Multimode Microplate Reader for time‐series OD_600_ measurements. A delay growth model was fitted to the time‐series OD_600_ measurements to infer the cell doubling time (Appendix S1 and S2).

### Flow cytometer measurement of SOS response in *E. coli* population

To characterize SOS response in *E. coli* population, cell cultures of different *E. coli* strains with the SOS response reporter plasmid pBbA6c_PsulA_sfGFP were diluted to OD_600_ 0.1 and cultured at 37°C with shaking (250 rpm) for 6 h. GFP expression was measured by the LSRFortessa X‐20 Flow Cytometer (BD Biosciences). A blue (488 nm) laser was used for GFP excitation, and a 530/30 nm filter was used for GFP emission. To quantify the percentage of SOS response‐activated cells, a threshold of GFP 10,000 (a.u.) was used to define the GFP ON subpopulation. Data analysis was performed using a customized MATLAB script. The distribution of GFP and Forward Scatter of single cells was plotted using FlowJo.

### Microscopic imaging of single cells

To image single cells of *E. coli* and *B. subtilis* in the co‐culture, an overnight culture of each strain was first diluted to OD_600_ 0.1 into LB supplemented with 50 mM xylose. Cells were co‐cultured for 3 h, and 4 μl of the cell culture was transferred to a glass slide. Five microliter 0.1% (w/v) Poly‐L‐lysine (Millipore Sigma) was spread evenly on the glass slide to allow the cells to adhere to the surface. Pipetting was avoided to prevent the disruption of the physically associated cells. A coverslip was gently placed on the culture on the glass slide.

Single cells were imaged by Ti‐E Eclipse inverted microscope (Nikon) with 40× magnification. GFP, RFP, and phase‐contrast images were taken from multiple spots on the glass slide. Filters used for GFP (Chroma) were 470/40 nm (excitation) and 525/50 nm (emission). Filters used for RFP (Chroma) were 560/40 nm (excitation) and 630/70 nm (emission). To image SOS response of single cells, cell culture of *E. coli* MG1655 with the SOS response reporter plasmid pBbA6c_PsulA_sfGFP and pBB275 or pUC19 was diluted to an OD_600_ 0.1 and cultured at 37°C with shaking (250 rpm) for 6 h. Four microliter cell culture was transferred to the glass slide and imaged by Nikon Eclipse Ti Microscope with 40× magnification. Phase‐contrast and GFP images were taken to determine the presence of filamentous and fluorescent cells in *E. coli* MG1655 harboring pBB275.

### Bioinformatic analysis of plasmid transfer to *Bacillus* and non‐*Bacillus* bacteria

To search *E. coli* plasmid replication origins in the Genus *Bacillus*, ColE1 (589 bp in pUC19, Addgene Plasmid #50005), p15A (712 bp in pBbA6c‐RFP, Addgene Plasmid #35290), CloDF13 (739 bp in pCSaE500, Addgene Plasmid #53182), and pSC101 (2,224 bp in pBbS2k‐RFP, Addgene Plasmid #35330) were searched in NCBI RefSeq Genome Database. Nucleotide BLAST was optimized for highly similar sequences. The organism was limited to *Bacillus* (taxid:1386). Max target sequences of 5,000 were selected. To search *E. coli* plasmid replication origins in non‐*Bacillus* bacteria, the organism was limited to Bacteria (taxid:2) and excluded *Escherichia* (taxid:561) and *Bacillus* (taxid:1386). Accession date of data is 2021‐09‐03. Hits were listed in Dataset EV1. Hits with > 90% identity and > 80% percentage were selected for the analysis of sequence length. Query coverage is the percentage of the query sequence aligned to the hit sequence. Percent identity is the percentage of nucleotides that match the alignment. A sequence length longer than 10^6^ bp suggests a potential genomic integration of plasmid. Shorter sequence lengths may be caused by the contamination of plasmid DNA during library preparation or sequencing steps (Wally *et al*, 2019). A phylogenetic tree was constructed by aligning 16S rRNA sequences using neighbor joining method in MEGA v10.2.6 (Kumar *et al*, 2018). To annotate genes near plasmid origin replication in *Bacillus* genomes, GenBank sequences were downloaded from NCBI website and analyzed using SnapGene Viewer v5.3.2.

## Author contributions

**Yu‐Yu Cheng:** Conceptualization; software; validation; investigation; visualization; methodology; writing – original draft; writing – review and editing. **Zhichao Zhou:** Resources; software; investigation; methodology. **James M Papadopoulos:** Resources; investigation. **Jason D Zuke:** Resources; investigation. **Tanya G Falbel:** Resources; validation; investigation; methodology. **Karthik Anantharaman:** Resources; software; investigation. **Briana M Burton:** Conceptualization; resources; supervision; validation; investigation; methodology; project administration; writing – review and editing. **Ophelia S Venturelli:** Conceptualization; resources; supervision; funding acquisition; validation; investigation; visualization; methodology; writing – original draft; project administration; writing – review and editing.

## Disclosure and competing interests statement

The authors declare that they have no conflict of interest.

## Supporting information



Appendix

Dataset EV1

Source Data for Figure 1

Source Data for Figure 2

Source Data for Figure 3

Source Data for Figure 4

## Data Availability

Nucleotide BLAST results of *E. coli* plasmid replication origins in bacterial genome are included in Dataset EV1. Source data of bacterial CFU, transformation frequency, HGT efficiency, and eDNA concentration in primary data can be downloaded from https://www.ebi.ac.uk/biostudies with the accession number S‐BSST1002.
