# Supplementary material for: Efficient plasmid transfer via natural competence in a microbial co‐culture
Source: Mol Syst Biol. 2023 Jan 30;19(3):e11406. doi: 10.15252/msb.202211406 (PMC9996237; doi:10.15252/msb.202211406)
Supplement: Supplementary file 1 — Appendix [file MSB-19-e11406-s006.pdf]

Supporting information

Efficient plasmid transfer via natural competence in a microbial co-culture

Yu-Yu Cheng[1], Zhichao Zhou[2], James M. Papadopoulos[3], Jason D. Zuke[2], Tanya G. Falbel[2], Karthik Anantharaman[2], Briana M. Burton[2], and Ophelia S. Venturelli[1,2,3*]

[1]Department of Biochemistry, University of Wisconsin - Madison, WI, United States
[2]Department of Bacteriology, University of Wisconsin - Madison, WI, United States
[3]Department of Chemical & Biological Engineering, University of Wisconsin-Madison

*To whom correspondence should be addressed: venturelli@wisc.edu

This file includes: **Page**

Description of the delay growth model. 2
Appendix Figure S1. Characterization of plasmid transfer in the co-culture. 3
Appendix Figure S2. Characterization of genomic DNA transfer in the co-culture. 4
Appendix Figure S3. Growth characterization of *recA*+ or *recA*- *E. coli* donors. 5
Appendix Figure S4. Plasmid maps for characterizing HGT efficiency, plasmid multimerization, and eDNA release in the co-culture. 5
Appendix Figure S5. Induction of the SOS response in *E. coli* MG1655 harboring pBB275. 6
Appendix Figure S6. Gel image of pBB275 plasmid in the co-culture supernatant. 7
Appendix Figure S7. Time-series measurements of eDNA release from live, heat-killed, or chloramphenicol treated *E. coli*. 7
Appendix Figure S8. Nucleotide BLAST search results to identify ColE1, p15A, pSC101, and CloDF13 plasmid replication origins in *Bacillus* and non-*Bacillus* DNA sequences. 8
Appendix Figure S9. Presence of ColE1 plasmid replication origin in *Bacillus* and non-*Bacillus* genomes. 9
Appendix Table S1. List of plasmids. 10
Appendix Table S2. List of bacterial strains. 10
Appendix Table S3. Sequences of qPCR primers. 12

Delay growth model for inferring doubling time from growth curve

To estimate the doubling time of *E. coli* from the growth curve, a coupled ordinary differential equation model was fit to the time-series measurements of absorbance at 600 nm (OD) (Fridman *et al*, 2014). The two species E_n and E_g represent the sub-populations of non-growing and growing cells, respectively. The equations for this growth model are

$$\frac{dE_n}{dt} = -\, k_g E_n, \qquad \text{S1}$$

$$\frac{dE_g}{dt} = \; k_g E_n + \mu_e E - \alpha_{ee} E^2 \;, \qquad \text{S2}$$

where k_g represents the transition rate from the non-growing sub-population E_n to growing sub-population E_g, μ_e denotes the growth rate, and α_{ee} represents the intra-species interaction coefficient (growth capacity) of the growing sub-population. We fit the model to the time-series OD measurements using custom codes in MATLAB (Venturelli *et al*, 2018). Briefly, a nonlinear programming solver was used to minimize the mean squared error between the model prediction and experimental measurement across all time points to estimate optimal parameter sets. The doubling time t_D in **Fig S3C** was computed using the equation $t_D = \log_e 2/\mu_e$.

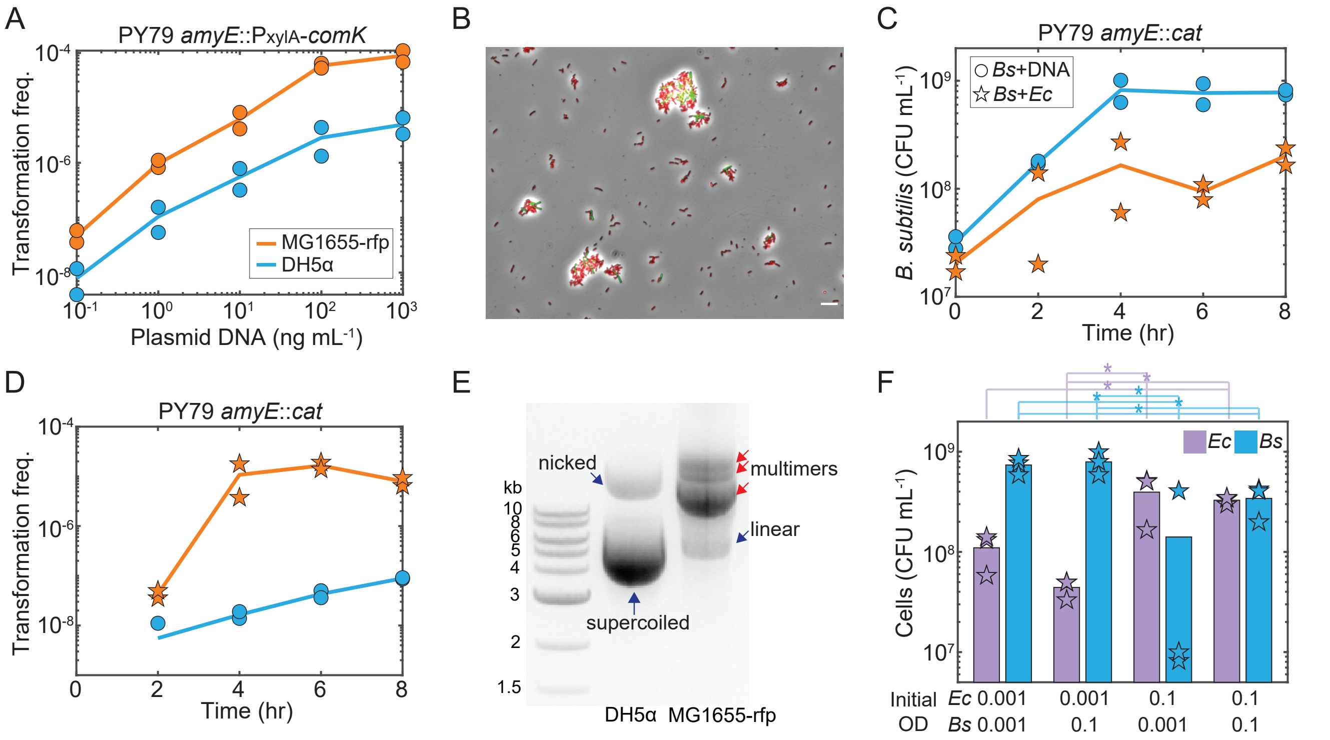

Appendix Figure S1. Characterization of plasmid transfer in the co-culture. (A) Scatter plot of the initial pBB275 plasmid concentration and transformation frequency in the engineered *B. subtilis* monoculture at 6 hr. Plasmid derived from *E.coli* MG1655-rfp displayed ~10-fold higher transformation frequency than plasmid derived from *E. coli* DH5α. **(B)** Microscopic image of *E. coli* MG1655-rfp (red) and engineered *B. subtilis* (green) in the microbial co-culture at 3 hr. Scale bar is 10 μm. Time-series measurements of **(C)** the abundance of engineered *B. subtilis* without xylose-inducible *comK* and **(D)** transformation frequency of the pBB725 plasmid in the monoculture or co-culture with *E. coli* MG1655-rfp harboring pBB275 in LB. In the monoculture, 100 ng/mL pBB275 plasmid DNA derived from *E. coli* DH5α was introduced. One biological replicate was below the detection limit (10^{-9} transformation frequency) at 2 hr. **(E)** Gel image of the pBB275 plasmid extracted from *E. coli* MG1655-rfp or DH5α. Plasmids extracted from *E. coli* MG1655-rfp contained multimers. Multimers had larger molecular weight than monomers (nicked, linear, and supercoiled indicated by blue arrows) and migrated slower on the gel during electrophoresis. **(F)** Bar plot of the abundances of *E. coli* MG1655-rfp harboring pBB275p plasmid or engineered *B. subtilis* at 6 hr in the co-culture inoculated with different initial species densities. Unpaired *t*-test was used to determine if the abundances between two conditions were statistically different. A star (*) indicates a statistical difference with *p*-value < 0.05. Low initial density of *E. coli* (OD0.001) yielded lower *E. coli* density and high *B. subtilis* at 6 hr, whereas high initial density of *E. coli* (OD0.1) yielded higher *E. coli* density and lower *B. subtilis* at 6 hr. DNA titration experiment in **(A)** and time-series experiment in **(C)** and **(D)** had two biological replicates. Initial cell density experiment in **(F)** had three biological replicates. Lines and bars are the average of the biological replicates.

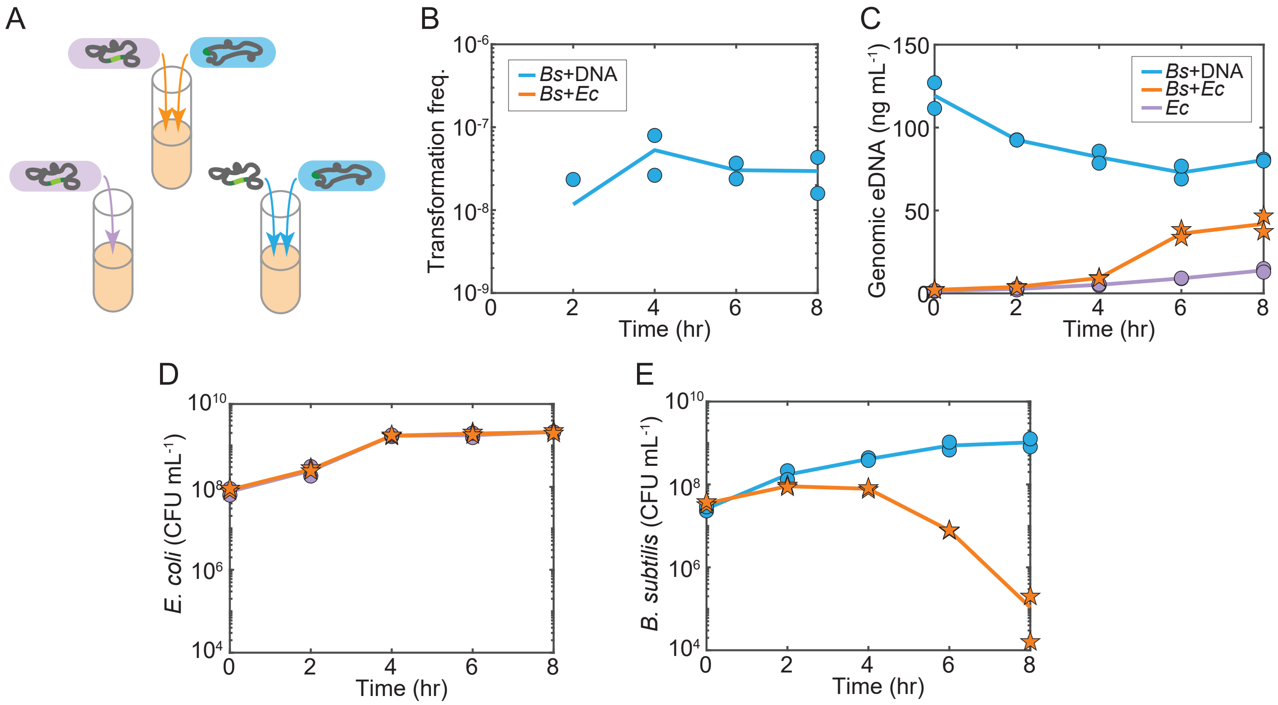

Appendix Figure S2. Characterization of genomic DNA transfer in the co-culture. (A) Schematic of experimental design to characterize the temporal changes in transfer of genomic DNA (gDNA), species abundance and extracellular gDNA release in *E. coli* monoculture (purple), *B. subtilis* monoculture supplemented with 100 ng/mL *E. coli* gDNA (blue) or co-culture composed of *E. coli* and *B. subtilis* (orange). *E. coli* MG1655 harbored a genomically integrated erythromycin resistance gene flanked by two ~600 bp *B. subtilis* PY79 *yvbJ*. *B. subtilis* was engineered to harbor a xylose-inducible master regulator for competence *comK* for enhancing transformation efficiency in the co-culture. Time-series measurements of **(B)** the transformation frequency of *E. coli* gDNA in monoculture or co-culture, **(C)** extracellular *E. coli* gDNA concentration, **(D)** *E. coli* abundance, and **(E)** *B. subtilis* abundance. Each experiment had two biological replicates. One biological replicate in **(B)** for *B. subtilis* monoculture transformation was below detection limit at 2 hr. Lines are the average of the biological replicates.

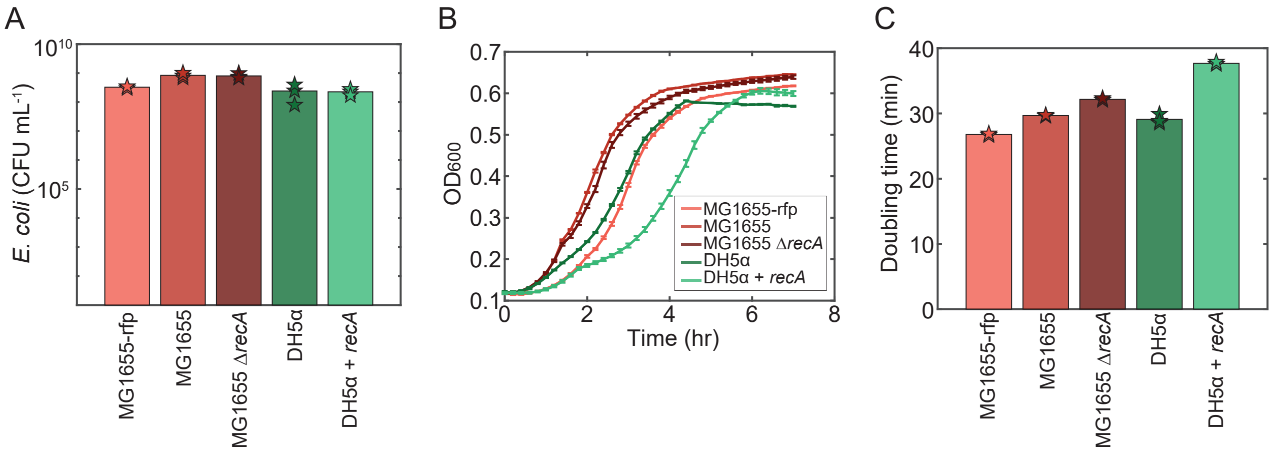

Appendix Figure S3. Growth characterization of *recA*+ or *recA*- *E. coli* donors. (A) Abundance of the *recA*+ or *recA*- *E. coli* plasmid donors at 6 hr in the co-culture with engineered *B. subtilis*. All *E. coli* strains harbored the pBB275 plasmid. **(B)** Time-series measurements of absorbance at 600 nm (OD_{600}) for the *recA*+ or *recA*- *E. coli* strains harboring pBB275 plasmid in monoculture. **(C)** Inferred doubling times of the *recA*+ or *recA*- *E. coli* strains based on the growth curve in (B) fit with a delay growth model. Bars and lines are the average of three biological replicates. Error bars in **(B)** denote 1 s.d. from the average.

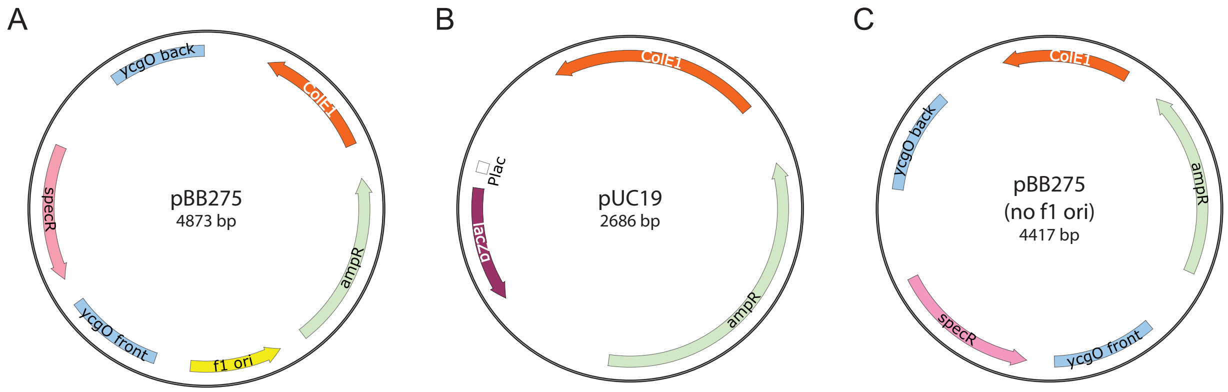

Appendix Figure S4. Plasmid maps for characterizing HGT efficiency, plasmid multimerization, and eDNA release in the co-culture. (A) Map of the pBB275 plasmid. *specR* denotes the spectinomycin resistance gene used for the selection of *B. subtilis* genomic integration in *ycgO* locus. The f1 ori denotes the phage replication origin, a remnant of the original plasmid with the ColE1 replication origin and ampicillin resistance gene *ampR*. **(B)** Map of the pUC19 plasmid. pUC19 plasmid contains the same ColE1 replication origin and *ampR* as the ones in pBB275 plasmid and a β-galactosidase gene *lacZα* from *E. coli lac* operon. **(C)** Map of the pBB275 plasmid lacking the f1 origin.

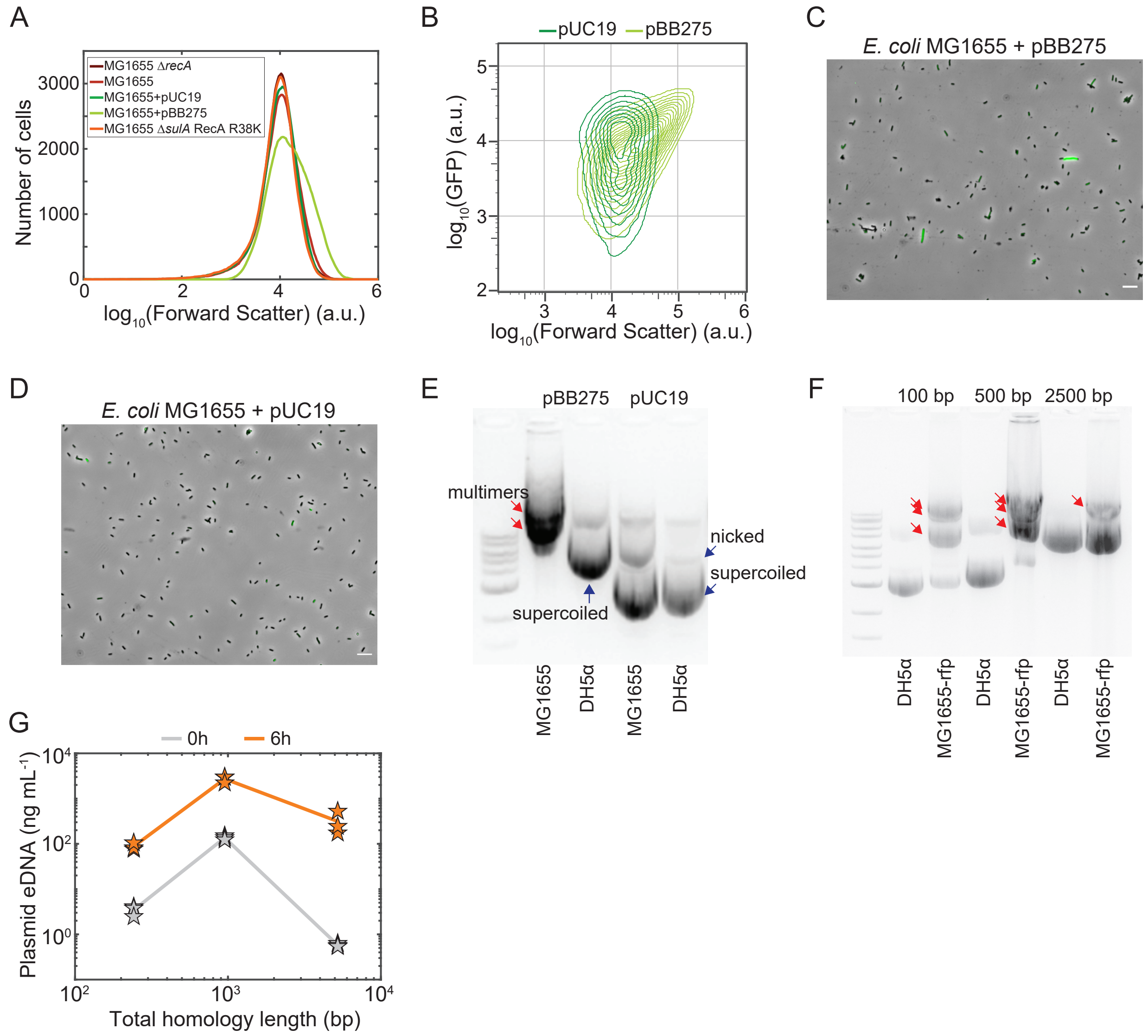

Appendix Figure S5. Induction of the SOS response in *E. coli* MG1655 harboring pBB275. **(A)** Histogram of forward scatter based on flow cytometry of *E. coli* MG1655 Δ*recA*, *E. coli* MG1655, *E. coli* MG1655 harboring pUC19, *E. coli* MG1655 harboring pBB275, or *E. coli* MG1655 Δ*sulA* RecA E38K at 6 hr in monoculture. All *E. coli* strains harbored SOS response reporter plasmid. **(B)** Contour plot of forward scatter and GFP expression of *E. coli* MG1655 harboring pUC19 or *E. coli* MG1655 harboring pBB275 at 6 hr in monoculture based on flow cytometry. **(C)** Microscopic image of *E. coli* MG1655 harboring pBB275 and SOS response reporter plasmid. Scale bar is 10 μm. **(D)** Microscopic image of *E. coli* MG1655 harboring pUC19 and SOS response reporter plasmid. Scale bar is 10 μm. **(E)** Gel image of pBB275 and pUC19 extracted from *E. coli* MG1655 or DH5α. A 1 kb DNA Ladder was used as a reference for DNA sizes. **(F)** Gel image of pBB275 plasmid with ~100 bp, ~500 bp, or ~2500 bp homology arms extracted from *E. coli* MG1655-rfp or DH5α. A 1 kb DNA Ladder was used for reference for DNA sizes. **(G)** Extracellular pBB275 plasmid concentration at 0 hr or 6 hr in the co-culture composed of *E. coli* MG1655-rfp harboring pBB275 and engineered *B. subtilis*. Plasmids with different homology arm lengths were constructed and tested. Experiments in **(A)**, **(B)**, and **(G)** had three biological replicates. Lines are the average of the biological replicates.

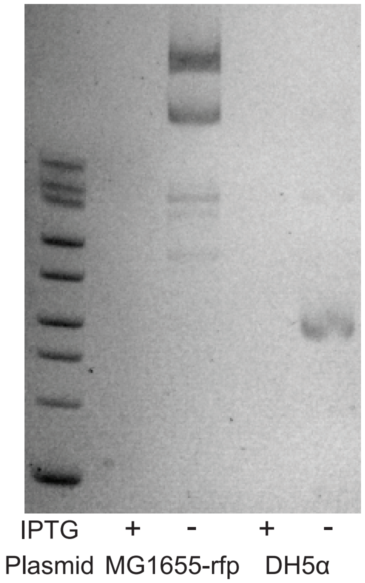

Appendix Figure S6. Gel image of pBB275 plasmid in the co-culture supernatant. The pBB275 plasmid was extracted from *E. coli* MG1655-rfp or DH5α and 1 μg/mL pBB275 plasmid DNA was externally introduced into the co-culture composed of engineered *B. subtilis* and *E. coli* harboring an IPTG-inducible lysis gene on a plasmid. The DNA lengths in the supernatant of co-culture was analyzed by gel electrophoresis. No DNA band was observed for co-culture with the addition of 0.2 mM IPTG for both multimeric and monomeric pBB275 plasmid. A 1 kb Extend DNA Ladder was used as a reference.

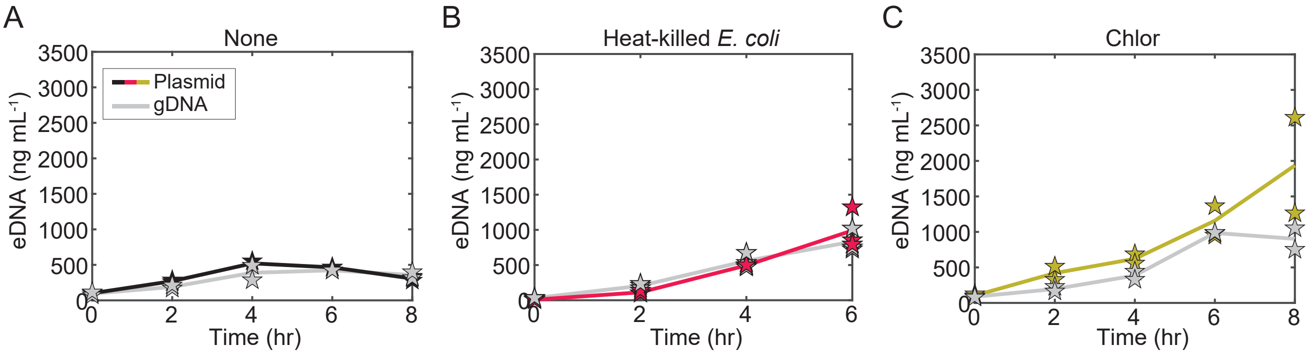

Appendix Figure S7. Time-series measurements of eDNA release live, from heat-killed, or chloramphenicol treated *E. coli*. Time-series measurements of extracellular pBB275 plasmid and *E. coli* gDNA concentrations of **(A)** live, **(B)** heat-killed, or **(C)** 5 μg/mL chloramphenicol treated *E. coli* MG1655-rfp harboring pBB275 in the co-culture with engineered *B. subtilis*. Live and chloramphenicol treated *E. coli* experiments in **(A)** and **(C)** had two biological replicates. The heat-killed *E. coli* experiment in **(B)** had three biological replicates. Lines are the average of the biological replicates. Note that the plasmid concentrations in **Appendix Fig S7A,C** are the same as **Fig 4G**. The data were included here for the comparison with extracellular gDNA concentrations.

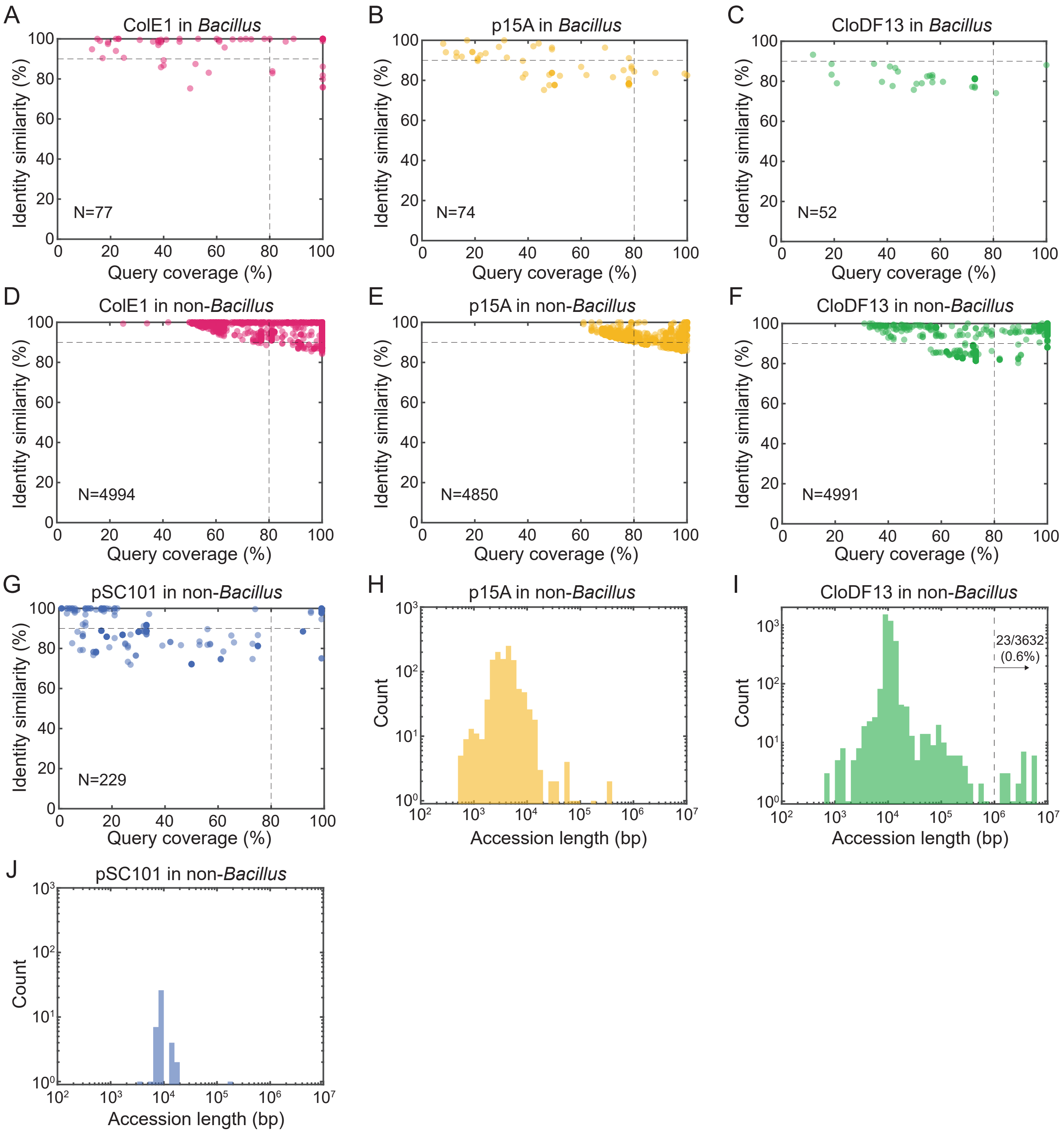

Appendix Figure S8. Nucleotide BLAST search results to identify ColE1, p15A, pSC101, and CloDF13 plasmid replication origins in *Bacillus* and non-*Bacillus* DNA sequences. (A) Scatter plot of query coverage and percent identity of ColE1 in 77 *Bacillus* DNA sequences in the NCBI RefSeq Genome Database. Hits with >90% identity and >80% coverage were selected for the analysis of sequence length. **(B)** Scatter plot of query coverage and percent identity of p15A in 74 *Bacillus* DNA sequences. **(C)** Scatter plot of query coverage and percent identity of CloDF13 in 52 *Bacillus* DNA sequences. **(D)** Scatter plot of query coverage and percent identity of ColE1 in 4994 non-*Bacillus* DNA sequences. **(E)** Scatter plot of query coverage and percent identity of p15A in 4850 non-*Bacillus* DNA sequences. **(F)** Scatter plot of query coverage and percent identity of CloDF13 in 4991 non-*Bacillus* DNA sequences. **(G)** Scatter plot of query coverage and percent identity of pSC101 in 229 non-*Bacillus* DNA sequences. Histograms of the sequence lengths of non-*Bacillus* DNA sequences containing **(H)** p15A, **(I)** CloDF13, or **(J)** pSC101 replication origin.

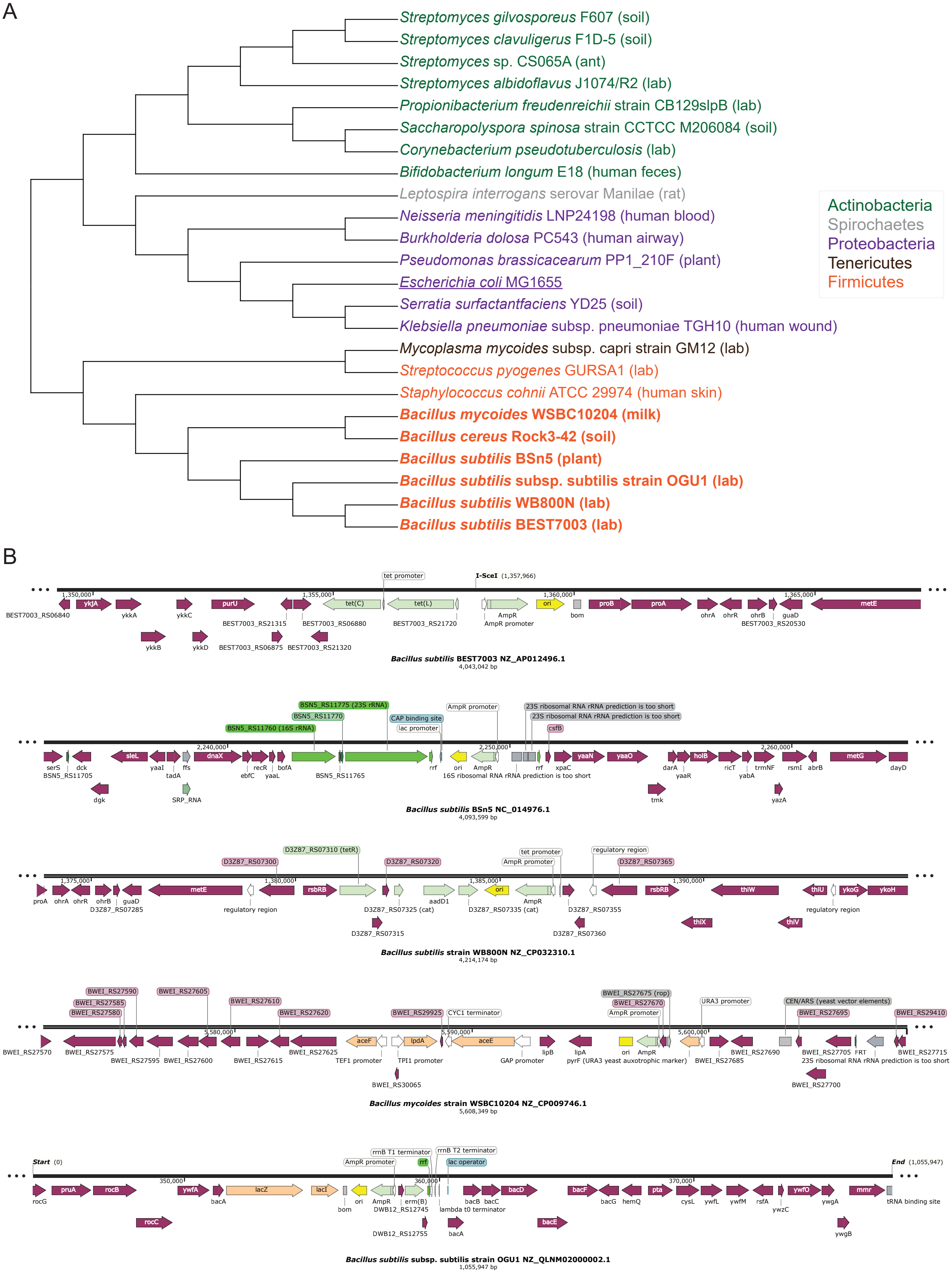
A
Streptomyces gilvosporeus F607 (soil)
Streptomyces clavuligerus F1D-5 (soil)
Streptomyces sp. CS065A (ant)
Streptomyces albidoflavus J1074/R2 (lab)
Propionibacterium freudenreichii strain CB129slpB (lab)
Saccharopolyspora spinosa strain CCTCC M206084 (soil)
Corynebacterium pseudotuberculosis (lab)
Bifidobacterium longum E18 (human feces)
Leptospira interrogans serovar Manilae (rat)
Neisseria meningitidis LNP24198 (human blood)
Burkholderia dolosa PC543 (human airway)
Pseudomonas brassicacearum PP1_210F (plant)
Escherichia coli MG1655
Serratia surfactantfaciens YD25 (soil)
Klebsiella pneumoniae subsp. pneumoniae TGH10 (human wound)
Mycoplasma mycoides subsp. capri strain GM12 (lab)
Streptococcus pyogenes GURSA1 (lab)
Staphylococcus cohnii ATCC 29974 (human skin)
Bacillus mycoides WSBC10204 (milk)
Bacillus cereus Rock3-42 (soil)
Bacillus subtilis BSn5 (plant)
Bacillus subtilis subsp. subtilis strain OGU1 (lab)
Bacillus subtilis WB800N (lab)
Bacillus subtilis BEST7003 (lab)
Actinobacteria
Spirochaetes
Proteobacteria
Tenericutes
Firmicutes
B
tet promoter
I-SceI (1,357,966)
1,350,000
1,355,000
1,360,000
1,365,000
ykjA
purU
tet(C)
tet(L)
ori
proB
proA
metE
BEST7003_RS06840
ykkA
ykkC
BEST7003_RS21315
BEST7003_RS06880
BEST7003_RS21720
AmpR
AmpR promoter
bom
ohrA
ohrR
ohrB
guaD
BEST7003_RS20530
ykkB
ykkD
BEST7003_RS06875
BEST7003_RS21320
Bacillus subtilis BEST7003 NZ_AP012496.1
4,043,042 bp
BSN5_RS11775 (23S rRNA)
BSN5_RS11770
BSN5_RS11760 (16S rRNA)
AmpR promoter
CAP binding site
lac promoter
23S ribosomal RNA rRNA prediction is too short
23S ribosomal RNA rRNA prediction is too short
csfB
2,240,000
2,250,000
2,260,000
sleL
dnaX
yaaN
yaaO
holB
metG
serS
dck
BSN5_RS11705
yaaI
tadA
ffs
recR
ebfC
yaaL
bofA
BSN5_RS11765
rrf
ori
AmpR
16S ribosomal RNA rRNA prediction is too short
xpaC
darA
yaaR
ricT
yabA
trmNF
rsmI
abrB
dayD
dgk
SRP_RNA
tmk
yazA
Bacillus subtilis BSn5 NC_014976.1
4,093,599 bp
D3Z87_RS07310 (tetR)
D3Z87_RS07300
D3Z87_RS07320
tet promoter
AmpR promoter
regulatory region
D3Z87_RS07365
1,375,000
1,380,000
1,385,000
1,390,000
metE
rsbRB
ori
rsbRB
thiW
thiU
ykoG
ykoH
proA
ohrA
ohrR
ohrB
guaD
D3Z87_RS07285
regulatory region
D3Z87_RS07325 (cat)
aadD1
D3Z87_RS07335 (cat)
AmpR
D3Z87_RS07355
regulatory region
thiX
thiV
D3Z87_RS07315
D3Z87_RS07360
Bacillus subtilis strain WB800N NZ_CP032310.1
4,214,174 bp
BWEI_RS27590
BWEI_RS27585
BWEI_RS27580
BWEI_RS27605
BWEI_RS27610
BWEI_RS27620
BWEI_RS29925
CYC1 terminator
BWEI_RS27675 (rop)
BWEI_RS27670
AmpR promoter
URA3 promoter
CEN/ARS (yeast vector elements)
BWEI_RS27695
BWEI_RS29410
5,580,000
5,590,000
5,600,000
aceF
lpdA
aceE
BWEI_RS27570
BWEI_RS27575
BWEI_RS27595
BWEI_RS27600
BWEI_RS27615
BWEI_RS27625
TEF1 promoter
TPI1 promoter
GAP promoter
lipB
lipA
pyrF (URA3 yeast auxotrophic marker)
ori
AmpR
BWEI_RS27685
BWEI_RS27690
BWEI_RS27705
FRT
BWEI_RS27715
23S ribosomal RNA rRNA prediction is too short
BWEI_RS30065
BWEI_RS27700
Bacillus mycoides strain WSBC10204 NZ_CP009746.1
5,608,349 bp
rrnB T1 terminator
rrnB T2 terminator
AmpR promoter
rrf
lac operator
Start (0)
End (1,055,947)
350,000
360,000
370,000
pruA
rocB
ywfA
lacZ
lacI
bacD
bacF
pta
ywfO
mmr
rocG
bacA
bom
ori
AmpR
DWB12_RS12745
erm(B)
lambda t0 terminator
bacB
bacC
bacG
hemQ
cysL
ywfL
ywfM
rsfA
ywzC
ywgA
tRNA binding site
rocC
DWB12_RS12755
bacA
bacE
ywgB
Bacillus subtilis subsp. subtilis strain OGU1 NZ_QLNM02000002.1
1,055,947 bp

Appendix Figure S9. Presence of ColE1 plasmid replication origin in *Bacillus* and non-*Bacillus* genomes. (A) Phylogenetic tree of bacteria that had a ColE1 replication origin identified in their genomes. *E. coli* was included to compare the evolutionary distances of bacterial strains. Different phyla are labeled with different colors. The environments where the bacteria were isolated are indicated. **(B)** Maps of the five *Bacillus* genomes where ColE1 was found. Antibiotic resistance genes (green) and heterologous genes (orange) can be found near the ColE1 replication origin. Note that *Bacillus cereus* Rock3-42 in **Dataset EV1** was excluded due to scaffold gaps near ColE1.

Appendix Table S1. List of plasmids.

Plasmid	Genotype	Description
pBB275_100bp	*ycgO*(150 bp)-*specR*-*ycgO*(92 bp), f1 ori, ColE1, *ampR*	Integrative plasmid for *B. subtilis* transformation with ~100 bp *ycgO* homology arms
pBB275_500bp	*ycgO*(475 bp)-*specR*-*ycgO*(477 bp), f1 ori, ColE1, *ampR*	Integrative plasmid for *B. subtilis* transformation with ~500 bp *ycgO* homology arms
pBB275_2500bp	*ycgO*(2636 bp)-*specR*-*ycgO*(2583 bp), f1 ori, ColE1, *ampR*	Integrative plasmid for *B. subtilis* transformation with ~2500 bp *ycgO* homology arms
pUC19	*lacZα*, ColE1, *ampR*	ColE1 plasmid without f1 origin and *B. subtilis* homology arms
pBB275 (no f1 ori)	*ycgO*(475 bp)-*specR*-*ycgO*(477 bp), ColE1, *ampR*	Integrative plasmid for *B. subtilis* transformation with ~500 bp *ycgO* homology arms and no f1 replication origin
pBbA6k_J23100_recA	P_{J23100}-*recA*, p15A, *kanR*	Constitutively expressed *recA*
pBbA6c_PsulA_sfGFP	P_{sulA}-*sfgfp*, p15A, *cat*	SOS response reporter
pYC01	$P_{A1lacO\text{-}1}$-*E*, CloDF13, *smR*	IPTG-inducible phage φX174 lysis gene *E*

Appendix Table S2. List of bacterial strains.

Strain	Genotype	Description	Figures
msOSV01034	*E. coli* MG1655 *caiE*::P_{J23100}-*mCherry* (pBB275_500bp)	*E. coli* MG1655-rfp plasmid donor with pBB275_500bp	1, 2A-C, 2H, 3I, 4, S1A, S1C-F, S3, S5F-G, S6, and S7
msOSV01033	*B. subtilis* PY79 *amyE*::P_{xylA}-*comK*, *cat*; *lacA*::*ermR*	Engineered *B. subtilis* for enhanced plasmid transformation in LB	1, 2A-C, 2E-F, 2H, 3, 4, S1A, S1F,

			S5G, S6, and S7
msOSV00858	*B. subtilis* PY79 *amyE*::*cat*; *lacA*::*ermR*	*B. subtilis* without xylose-inducible *comK* for plasmid transformation	S1C-D
msOSV00389	*E. coli* DH5α (pBB275_500bp)	*E. coli* DH5α plasmid donor with pBB275_500bp	1B-F, 2A-B, 2H, 3I, S1A, S1C-E, S3, S5E-F, and S6
msOSV01032	*B. subtilis* PY79 *comEC*::*kanR*; *amyE*::P_{xylA}-*comK*, *cat*; *lacA*::*ermR*	Engineered *B. subtilis* without *comEC*	1F
msOSV00348	*E. coli* MG1655 *caiE*::*yvbJ*(595 bp)-*ermR*-*yvbJ*(594 bp)	*E. coli* MG1655 gDNA donor	S2
msOSV00184	*B. subtilis* PY79 *amyE*::P_{xylA}-*comK*, *cat; yhdGH*::*kanR*	Engineered *B. subtilis* for *E. coli* gDNA transformation	S2
msOSV01107	*E. coli* MG1655 (pBB275_500bp)	*E. coli* MG1655 plasmid donor with pBB275_500bp	2A-C, 2E-F, S3, and S5E
msOSV01105	*E. coli* MG1655 Δ*recA* (pBB275_500bp)	*E. coli* MG1655 Δ*recA* plasmid donor with pBB275_500bp	2A-C and S3
msOSV01106 *	*E. coli* DH5α (pBB275_500bp, pBbA6k_J23100_recA)	*E. coli* DH5α+*recA* plasmid donor with pBB275_500bp and pBbA6k_J23100_recA	2A-B and S3
msOSV01115	*E. coli* MG1655 Δ*recA* (pBbA6c_PsulA_sfGFP)	*E. coli* MG1655 Δ*recA* with SOS response reporter plasmid	2D and S5A
msOSV01116	*E. coli* MG1655 (pBbA6c_PsulA_sfGFP)	*E. coli* MG1655 with SOS response reporter plasmid	2D and S5A
msOSV01141	*E. coli* MG1655 (pBbA6c_PsulA_sfGFP, pUC19)	*E. coli* MG1655 with SOS response reporter plasmid and pUC19 plasmid	2D, S5A-B, and S5D
msOSV01119	*E. coli* MG1655 (pBbA6c_PsulA_sfGFP, pBB275_500bp)	*E. coli* MG1655 with SOS response reporter plasmid and pBB275_500bp plasmid	2D and S5A-C
msOSV01124	*E. coli* MG1655 Δ*sulA* RecA E38K::*kanR* (pBbA6c_PsulA_sfGFP)	*E. coli* MG1655 Δ*sulA* RecA E38K with SOS response reporter plasmid and constitutive strong SOS response	2D and S5A
msOSV01138	*E. coli* MG1655 (pUC19)	*E. coli* MG1655 with pUC19 plasmid	2E and S5E
msOSV01139	*E. coli* DH5α (pUC19)	*E. coli* DH5α with pUC19 plasmid	S5E
msOSV01254	*E. coli* MG1655 (pBB275 (no f1 ori))	*E. coli* MG1655 plasmid donor with pBB275 (no f1 ori) plasmid	2F

msOSV01036	*E. coli* MG1655 *caiE*::P_{J23100}-*mCherry* (pBB275_100bp)	*E. coli* MG1655-rfp plasmid donor with pBB275_100bp	2H and S5F-G
msOSV01037	*E. coli* MG1655 *caiE*::P_{J23100}-*mCherry* (pBB275_2500bp)	*E. coli* MG1655-rfp plasmid donor with pBB275_2500bp	2H and S5F-G
msOSV00387	*E. coli* DH5α (pBB275_2500bp)	*E. coli* DH5α plasmid donor with pBB275_2500bp	2H and S5F
msOSV00388	*E. coli* DH5α (pBB275_100bp)	*E. coli* DH5α plasmid donor with pBB275_100bp	2H and S5F
msOSV01050	*E. coli* BW29427 (pBB275_100bp)	DAP-auxotrophic *E. coli* plasmid donor with pBB275_100bp	2I
msOSV01051	*E. coli* BW29427 (pBB275_500bp)	DAP-auxotrophic *E. coli* plasmid donor with pBB275_500bp	2I
msOSV01030	*E. coli* BW29427 (pBB275_2500bp)	DAP-auxotrophic *E. coli* plasmid donor with pBB275_2500bp	2I
msOSV00150	*B. subtilis* 168	Wild-type *B. subtilis* 168	2I
msOSV00117	*B. subtilis* natto IFO3335	Wild-type *B. subtilis* natto IFO3335	2I
usOSV00230	*B. subtilis* PY79	Wild-type *B. subtilis* PY79	2I
msOSV00608	*E. coli* MG1655 *caiE*::P_{J23100}-*mCherry* (pBB275_500bp, pYC01)	*E. coli* MG1655-rfp plasmid donor with pBB275_500bp and IPTG-inducible lysis gene plasmid	3A-H
msOSV01027	*E. coli* MG1655 *caiE*::P_{J23100}-*mCherry* (pYC01)	*E. coli* MG1655-rfp with IPTG-inducible lysis gene plasmid	3I and S6
msOSV00105	*E. coli* MG1655 *caiE*::P_{J21300}-*mCherry*	RFP-labeled *E. coli* for single-cell imaging	3I, S1B
msOSV00837	*B. subtilis* PY79 *ycgO*::$P_{hyperspank}$-*gfp*(Sp), *cat*; *lacA*::P_{xylA}-*comK*, *ermR*	GFP-labeled *B. subtilis* for single-cell imaging	S1B

* Kanamycin was not used in the preculture of *E. coli* since it impacted *B. subtilis* transformation in the co-culture.

Appendix Table S3. Sequences of qPCR primers.

Part	Sequence
pBB275(*specR*)_FW	CCCTATGTTCTAATGGAGAAGATTCA
pBB275(*specR*)_RV	ATCAGGATGATGAAACCAACTCT
pBB275(*specR*)_Probe	/56-FAM/AGATATTGC/ZEN/GGGAAATGCAGTGGC/3IABkFQ/
pUC19(*ampR*)_FW	CCCAACTGATCTTCAGCATCTT
pUC19(*ampR*)_RV	TTTCCGTGTCGCCCTTATTC
pUC19(*ampR*)_Probe	/56-FAM/ACTTTCACC/Zen/AGCGTTTCTGGGTGA/3IABkFQ/

E. coli MG1655(*caiE*::*ermR*)_FW	GGTTGATAATGAACTGTGCTGAT
E. coli MG1655(*caiE*::*ermR*)_RV	CGCATCCGATTGCAGTATAAAT
E. coli MG1655(*caiE*::*ermR*)_Probe	/56-FAM/CATCATGTT/ZEN/CATATTTATCAGAGCTCGTGC/3IABkFQ/
E. coli MG1655(*caiE*)_FW	ATACGCGGCGTCTTTCTTAC
E. coli MG1655(*caiE*)_RV	AGTGTCAGCGACTGGTTAAAG
E. coli MG1655(*caiE*)_Probe	/56-FAM/ATCGATGAG/ZEN/CAAATTTACGCGCGC/3IABkFQ/

References

Fridman O, Goldberg A, Ronin I, Shoresh N & Balaban NQ (2014) Optimization of lag time underlies antibiotic tolerance in evolved bacterial populations. *Nature* 513: 418–421

Venturelli OS, Carr A V, Fisher G, Hsu RH, Lau R, Bowen BP, Hromada S, Northen T & Arkin AP (2018) Deciphering microbial interactions in synthetic human gut microbiome communities. *Mol Syst Biol* 14: e8157
