# Supplementary material for: Efficient plasmid transfer via natural competence in a microbial co‐culture
Source: Mol Syst Biol. 2023 Jan 30;19(3):e11406. doi: 10.15252/msb.202211406 (PMC9996237; doi:10.15252/msb.202211406)
Supplement: Supplementary file 7 [file MSB-19-e11406-s004.pdf]

molecular systems biology

Efficient plasmid transfer via natural competence in a microbial co-culture

Yu-Yu Cheng, Zhichao Zhou, James Papadopoulos, Jason Zuke, Tanya Falbel, Karthik Anantharaman, Briana Burton, and Ophelia Venturelli
DOI: 10.15252/msb.202211406

Corresponding author(s): Ophelia Venturelli (venturelli@wisc.edu)

Review Timeline:

Submission Date:	13th Oct 20
Editorial Decision:	12th Nov 20
Resubmission Received:	17th Oct 22
Editorial Decision:	28th Nov 22
Revision Received:	21st Dec 22
Accepted:	9th Jan 23

Editor: Maria Polychronidou

Transaction Report:

(Note: With the exception of the correction of typographical or spelling errors that could be a source of ambiguity, letters and reports are not edited. Depending on transfer agreements, referee reports obtained elsewhere may or may not be included in this compilation. Referee reports are anonymous unless the Referee chooses to sign their reports.)

Thank you again for submitting your work to Molecular Systems Biology. We have now heard back from the three referees who agreed to evaluate your study. As you will see below, the reviewers raise substantial concerns on your work, which unfortunately preclude its publication in Molecular Systems Biology.

The reviewers appreciate the relevant topic and the extensive amount of data collected. However, they point out that as is stands the main conclusions remain unclear, and at times not well supported by the data and preformed analyses. During our cross-commenting process in which the reviewers are given the opportunity to comment on each other's reports, the three reviewers concluded that the concerns raised do not seem addressable within the scope of a major revision. As such they indicated that they do not support publication in Molecular Systems Biology.

Taken together and given the substantial concerns raised by the reviewers, I am afraid I see no other choice than to return the manuscript with the message that we cannot offer to publish it. I am sorry that the review of your work did not result in a more favorable outcome on this occasion, but I hope that you will not be discouraged from submitting future work to Molecular Systems Biology. In any case, thank you for the opportunity to examine this work.

Reviewer #1:

Cheng et al sought to investigate the role of donors on transformation efficiencies. To do so they measured the transformation efficiency of B. subtilis using a synthetic community of E. coli and B. subtilis with either plasmid or chromosomal DNA. They show that in co-culture the efficiency increases for plasmid and decreases for chromosomal DNA, and that it depends on donor presence. Overall, the question is quite interesting, and I commend the authors on the impressive amount of data collection and quantification. However, many aspects detract from the appeal of this paper. There are so many disjointed experimental components, the logic of many experiments was not clearly laid out, and the conclusions disregard many potential alternative explanations. This is likely amplified since the authors messaging is not clear, and the results are buried beneath dense and difficult to interpret figures. As someone who is extremely well-versed in the field of HGT, after reading the paper 4 or 5 times, I still could not state what the main findings were. Yes, clearly something about having the donor there changes the efficiency - but what is it due to? There is a fundamental difference between the donor actively changing the efficiency kinetics, versus the donor changing the recipient dynamics and thus indirectly altering the efficiency kinetics, and to me the study did not clearly differentiate the two; yet, this distinction drastically alters the conclusions. Thus, despite the extensive quantification, the rigor is not yet there. In addition to this major point, below are additional questions/comments:

1. It seems that the authors found so many interesting and significant findings, but they do not all belong in the same story - for example, the authors frame this manuscript as investigating the role of donor and recipients, but then focus on plasmid vs chromosomal DNA. Additionally, in line 52 "However...." The authors outline yet another open question that this paper is attempting to address (metabolic burden), which is different from both the donor/recipient data as well as the DNA type. The data presented does not sufficiently address all of these aspects; so, what is the main message the authors wished to focus on?

2. Why use different resistance genes for chromosomal vs plasmid DNA, when the experiments were done separately? The plasmid to chromosomal differences could potentially be explained by this; though unlikely, in the interest of rigor, the authors should switch the spec and ery genes to verify.
3. Results of paragraph 1 are very confusing to follow (lines 80-84). After reading several times, I understood the authors were using purified DNA in mono culture and E coli in co-culture, but the presentation was extremely dense. Consider breaking up the sentence, including "respectively", and any other means to clarify what exactly is being done. Likewise, even the figure 2 was not very clear and did not really help me interpret what the authors were doing.
4. It was never explained exactly how the eDNA would be released from E. coli, whether this was a stochastic or a constant expected process, and how delivery of purified plasmid and chromosomal DNA compare to the continuous co-culture setup. Why did the authors not use mono culture of E. coli to isolate and deliver throughout the time course, or multiple continuous delivery, like in the co-culture, instead of one bolus delivery at the beginning? The two methods are not necessarily comparable, and introduce a host of confounding factors into the primary setup that make it quite challenging to interpret.
5. It looks like the E. coli growth rate is different depending on whether it has the plasmid or chromosomal DNA version? Specifically, it looks like E coli grows faster in 2F then 2D. Did the authors quantify this and take it into account? Also, Bacillus grew more slowly in the presence of E. coli - but we do not know how growth impacts the transfer efficiency? Did the authors perform experiments that modulate the growth rate of Bacillus but without co-culture? This points back to confusions between donor actively changing the efficiency kinetics or not, and overall lack of clarity in the conclusions.
6. Lines 120-121, not nearly enough information about the filter separation experiment is given! "Our controls showed that 0.01% of E. coli were observed in the B. subtilis compartment at 6 hr, demonstrating the filter functions as a physical barrier". 0.01% of a dense culture is quite high. More importantly, they don't include the monoculture without the filter on the same graph (Fig. S2), which is the control of comparison here.
7. I did not understand the purpose of this spatial distribution section and the fluorescence microscopy sections. The quantification by looking at average ROI and cell aggregation did not contribute to the overall story, and made it even more confusing to interpret in the context of the main conclusions. Likewise, the rationale for focusing on plasmid metabolic burden, and how it connects to chromosomal transfer, was not explained.
8. "The plasmid transfer frequency was positively correlated with E. coli abundance (Pearson correlation coefficient r = 0.78, p-value = 9.37e-7), consistent with our model's prediction (Fig. 5 (A and B)). By contrast, the Ec-C abundance was not correlated to transformation frequency, mirroring the model prediction (Fig. 5, C and D)." While true, the y-axes are entirely different magnitudes, across most of the modeling in fact, which make the conclusions suspect especially in systems like these where biphasic or higher non-linearity would be observed. Could these simply be explained by the two operating in entirely different ranges? Can the authors artificially increase or decrease plasmid/chromosomal setup to operate in a comparable range? This again contributes to the major confusions outlined above between donor actively changing the efficiency kinetics or not.
9. The authors included dead E. coli in the model but not dead Bacillus. It wasn't clear if the dead E. coli was specifically when the lysis protein was expressed or just naturally as a contributor to eDNA. If the latter, dead Bacillus should also be included, in which case, how does it change the observed dynamics?

Reviewer #2:

In the current paper, the authors investigate competence-dependent HGT between E. coli as donor and B. subtilis as recipient. The premise of the study is in principle interesting but the experiments are not very well thought out, which is a pity as it renders the modelling part of the paper meaningless. Several key concepts about competence are not introduced and are not taken into account when designing the experiments. For instance, during competence dependent transformation, only a single strand of the transforming double stranded DNA enters the cell and is used for recombination. Where exactly the nick is made in the dsDNA donor is random. Therefore, the longer the tDNA fragment, the higher the transformation efficiency will be as the chance that this nick is made inside the homology region becomes smaller. However, when there is a lot of non-homologous DNA present, such as in the here used Ec-C case, then this will reduce transformation efficiency as there are only so many DNA uptake and recombination machineries per cell. It has also been shown before that transformation with multimeric DNA is much more efficient than with monomeric DNA. Modern miniprep kits mainly select for monomeric plasmids. It is not tested whether with the particular Ec-p plasmid this also influences HGT.

Specific comments:
L97: on the basis of the provided data, I do not think you can say the data demonstrates that inter-species interactions were a critical determinant of HGT efficiency. Plasmids directly coming from cells will also have plasmid multimers which transform much more efficient than plasmid isolated using kits (glass columns) which is typically more supercoiled.
For instance, monomeric plasmid transforms 1000 fold less efficient B. subtilis than multimeric plasmids (https://link.springer.com/article/10.1007/BF00267617).
L107: I do not think there is a causal relationship between reduced HGT with chromosomal DNA in the presence of the donor E. coli. This might simply show that there are less available eDNA molecules available to competent B. subtilis during co-culturing.
L200, increased E. coli lysis might not only induce DNA degradation, it will effectively also increase the pool of competing non-homologous DNA by the release of the other plasmid and chromosomal E. coli DNA. Having more DNA without homology regions to B. subtilis will decrease transformation efficiency.
L227: I think this experiment shows that with more available homologous DNA, transformation efficiency goes up. On the basis of the available data, I would not make a causal connection between plasmid metabolic burden and chromosomal HGT efficiency.
L314: the reported data does not challenge the current paradigm of competence-dependent transformation.
L319: My interpretation of the data is that if there is a higher concentration of homologous transforming DNA (when more donor E. coli cells lyse) and more viable competent recipient cells present (when B. subtilis is not dying), HGT rates are highest. When there is more competing non-transformable DNA present, transformation frequencies become lower. This is not a paradigm shift.

Reviewer #3:

In their manuscript, "Donor-recipient interactions drive dynamics of horizontal gene transfer via natural competence", Cheng et al. investigate the contributions of cell-cell interactions, DNA origin, and metabolic burden of plasmid carriage to the frequency of horizontal gene transfer using model

organisms E. coli and B. subtilis. The authors found, through experiments and modeling, that the rate of DNA transfer from the donor chromosome increased with increased abundance of recipient cells and increased lysis rate of donor cells. Additionally, the metabolic burden of plasmids increased bactericidal antibiotic susceptibility in donor strains, resulting in reduced transformation of plasmids to the recipient strain. Conversely, transfer of chromosome components increased in the presence of the same antibiotic due to alleviation of E. coli-induced suppression of B. subtilis growth (an increase in recipient strain abundance).
Interestingly, this work suggests that the availability of extracellular DNA is not the limiting factor controlling the rate of HGT. I appreciate the modeling and testing that was performed in conjunction with one another. It is difficult to say how applicable it will be to a real community, especially given that the metabolic burden, transfer rates, lysis susceptibility etc. will all be different, but it is interesting nonetheless.

Lines 96-98: It is unclear from your text whether "native comK B. subtilis PY79" is different from your xylose-inducible strain. The figure labels for figure S1 say "+ xylose / - xylose". Does this experiment represent two strains of B. subtilis (WT and xylose-inducible comK construct), or just the engineered strain with and without the inducer? If only the inducible comK strain was used for these experiments, was a qPCR or similar assay done to demonstrate a lack of leaky expression from the uninduced promoter?

Lines 141-143: Comparing the first panel of Figs. S3A and S3B, it looks like there is a greater proportion of GFP+ cells in the co-cultured experiment at the first time point. This is corroborated by Fig. S3C. It looks like the proportion of GFP+ cells are ~2.5X greater in the co-cultured experiment, though the lines converge for the monoculture and co-culture experiments in later time points. This is used to justify that there is no difference in competence activation for the B. subtilis cells in co-culture or monoculture. However, since comK is the master regulator, is it possible that the increased expression of this regulator in the first timepoint pays dividends in the expression of downstream com genes at later timepoints, leading to an appreciable difference in competence between treatments in the absence of a sustained difference in comK? Additionally, the literature suggests that B. subtilis regulates cellular levels of ComK proteolytically as well as transcriptionally. If we assume steady-state expression comK transcript, changes in the amount ClpC/MecA complex could lead to differences in the rate of ComK protein abundance and therefore differences in competence between groups.

Lines 152-156: The filter-based experiments showing that plasmid transfer is happening by free plasmid transformation rather than conjugation are convincing. However, are there differences in plasmid DNA topology between plasmids released in vitro (likely supercoiled) and isolated plasmid DNA (likely a mix of supercoiled and non-supercoiled species). Is there a difference in transformation efficiency of supercoiled and non-supercoiled plasmids that could explain, at least in part, the noted difference in B. subtilis competence in the co-culture and monoculture treatments?

Point-by-point response to reviewer comments:

Reviewer #1:

1. It seems that the authors found so many interesting and significant findings, but they do not all belong in the same story - for example, the authors frame this manuscript as investigating the role of donor and recipients, but then focus on plasmid vs chromosomal DNA. Additionally, in line 52 "However...." The authors outline yet another open question that this paper is attempting to address (metabolic burden), which is different from both the donor/recipient data as well as the DNA type. The data presented does not sufficiently address all of these aspects; so, what is the main message the authors wished to focus on?

In our new manuscript, we focused on studying the mechanisms determining plasmid HGT because we found that HGT efficiency is significantly higher in co-culture than in monoculture (~100-fold). We narrowed the focus of the manuscript to prioritize an understanding of the molecular mechanism and thus moved the chromosomal DNA data to supplementary information (Supplementary Fig. 2). We have identified the molecular mechanisms that enable high HGT efficiency in the community: the plasmid can become multimerized and enhances eDNA release via the induction of the *E. coli* donor strain SOS response (Fig. 2). We then investigated how different ecological factors could affect the efficiency of plasmid HGT (Fig. 3,4). We found that intact donor can lead to efficient HGT but counterintuitively not the lysed donor. Overall, we systematically investigated plasmid HGT via natural transformation and identified the critical molecular and ecological factors influencing HGT efficiency.

2. Why use different resistance genes for chromosomal vs plasmid DNA, when the experiments were done separately? The plasmid to chromosomal differences could potentially be explained by this; though unlikely, in the interest of rigor, the authors should switch the spec and ery genes to verify.

We tested different antibiotic resistance genes and integration loci and found that HGT was not affected by either different genes or integration loci. Since the chromosomal gene transfer is not the focus of the current paper, we moved the data of chromosomal gene transfer to the supplementary information (Fig. S2).

4. It was never explained exactly how the eDNA would be released from E. coli, whether this was a stochastic or a constant expected process, and how delivery of purified plasmid and chromosomal DNA compare to the continuous co-culture setup.

In our revised paper, we found that plasmid can induce SOS response and enhance eDNA release (Fig. 2d,e), indicating that the interplay of plasmid and host cell is a critical factor for eDNA release.

5. Why did the authors not use mono culture of E. coli to isolate and deliver throughout the time course, or multiple continuous delivery, like in the co-culture, instead of one bolus delivery at the beginning? The two methods are not necessarily comparable, and introduce a host of confounding factors into the primary setup that make it quite challenging to interpret.

By supplementing DNA in the beginning of the *B. subtilis* monoculture, we noticed that the eDNA concentration increased slightly over time in *B. subtilis* monoculture (Fig. 1d). This

indicates that the eDNA was not degraded or consumed by *B. subtilis* significantly over the course of 8 hours. Therefore, supplementing DNA at later times should not significantly affect transformation efficiency in the presence of saturated DNA concentrations.

5. It looks like the E. coli growth rate is different depending on whether it has the plasmid or chromosomal DNA version? Specifically, it looks like E coli grows faster in 2F then 2D. Did the authors quantify this and take it into account? Also, Bacillus grew more slowly in the presence of E. coli - but we do not know how growth impacts the transfer efficiency? Did the authors perform experiments that modulate the growth rate of Bacillus but without co-culture? This points back to confusions between donor actively changing the efficiency kinetics or not, and overall lack of clarity in the conclusions.

In our revised manuscript, we observed that growth rate of *E. coli* could not predict the HGT efficiency (Fig. 2a,b, Fig. 5e,h, and Supplementary Fig. 2b,c). Specifically, the doubling time was similar across all characterized *E. coli* strains (28-32 min), with the exception of the DH5a+recA (overexpression of RecA) strain which displayed a doubling time of 37 min (Fig. S3c). While *E. coli* MG1655 and MG1655 ΔrecA have similar doubling time, but HGT efficiency displayed a 100-fold difference (Fig. 2a, S3c). In addition, the slowest growing strain DH5a+recA displayed 100-fold higher HGT efficiency than DH5a, which displayed a faster doubling time (Fig. 2a). These data suggest that growth rate of *E. coli* is not a major determinant of HGT efficiency in our community.

In addition, we performed an experiment to introduce an antibiotic that directly inhibited *E. coli* growth and indirectly enhanced *B. subtilis* growth as a consequence of reduced ecological competition with *E. coli*. The results indicate that HGT efficiency was not substantially changed by antibiotic-induced perturbations on the growth rate of either species (Fig. 4d-i). We found that the critical factor influencing eDNA release and HGT efficiency is the plasmid-induced strong SOS response in a subpopulation of *E. coli* (Fig. 2d-g and Supplementary Fig. 5a-e).

9. The authors included dead E. coli in the model but not dead Bacillus. It wasn't clear if the dead E. coli was specifically when the lysis protein was expressed or just naturally as a contributor to eDNA. If the latter, dead Bacillus should also be included, in which case, how does it change the observed dynamics?

In our new manuscript, we investigate whether dead cells can contribute to eDNA release by killing the *E. coli* donor at 60 °C and supplementing these intact cells into a *B. subtilis* monoculture. Our new data show that dead cells can still release eDNA and achieve efficient HGT (Fig. 4a-c). Since the mechanism was more complex involving different molecular factors, developing a predictive mathematical model would require measuring many different parameters of the system, which is beyond the scope of this study. In addition, we find a simple factor *recA* that can explain most of our data without assumptions of underlying biochemical reactions in a model.

Reviewer #2:

L97: on the basis of the provided data, I do not think you can say the data demonstrates that inter-species interactions were a critical determinant of HGT efficiency. Plasmids directly coming

from cells will also have plasmid multimers which transform much more efficient than plasmid isolated using kits (glass columns) which is typically more supercoiled.
For instance, monomeric plasmid transforms 1000 fold less efficient B. subtilis than multimeric plasmids (https://link.springer.com/article/10.1007/BF00267617).

We compared the transformation efficiency of monomeric versus multimeric plasmid and found that multimeric plasmid has ~10-folds higher transformation efficiency than monomeric plasmid (Supplementary Fig. 1a). The plasmid miniprep kit we used did not remove the multimeric plasmid during the DNA extraction steps (Supplementary Fig. 1e). These results indicate plasmid conformation is one critical factor for efficient HGT. We also show that the SOS response in the donor is another critical factor to the eDNA release and HGT efficiency (Fig. 2d,e). Moreover, we found that eDNA release is enhanced in co-culture (Fig. 1e,2e), demonstrating that interspecies interaction is also important for HGT via natural transformation.

L107: I do not think there is a causal relationship between reduced HGT with chromosomal DNA in the presence of the donor E. coli. This might simply show that there are less available eDNA molecules available to competent B. subtilis during co-culturing.

We quantified the eDNA release of *E. coli* chromosome in the co-culture and the concentration was below an effective DNA concentration for transformation (Supplementary Fig. 2b,c). We agreed with the reviewer and modified the manuscript accordingly.

L200, increased E. coli lysis might not only induce DNA degradation, it will effectively also increase the pool of competing non-homologous DNA by the release of the other plasmid and chromosomal E. coli DNA. Having more DNA without homology regions to B. subtilis will decrease transformation efficiency.

We measured the gDNA release for the lysis experiment in addition to the plasmid release (Fig. 3c-f). Our results show *E. coli* can release a high amount of eDNA including plasmid and genomic DNA (Fig. 3c-f). To investigate whether gDNA could compete with plasmid for transformation, we supplemented 1 μg/mL *E. coli* gDNA to the *B. subtilis* monoculture containing 1 μg/mL *E. coli* plasmid DNA, which indeed reduced transformation efficiency of plasmid (Fig. 3i). In our new manuscript, we include both the DNase and gDNA effect on plasmid transformation (Fig. 3i and Supplementary Fig. 6).

L319: My interpretation of the data is that if there is a higher concentration of homologous transforming DNA (when more donor E. coli cells lyse) and more viable competent recipient cells present (when B. subtilis is not dying), HGT rates are highest. When there is more competing non-transformable DNA present, transformation frequencies become lower. This is not a paradigm shift.

We varied the initial OD of both *E. coli* and *B. subtilis* and found that eDNA release and HGT efficiency increased with the initial density of both species (Fig. 1g-i), consistent with the reviewer's prediction. In addition, we found that an optimal lysis rate and HGT efficiency is achieved through the induction of SOS response, and further increase in the lysis rate led to a decrease in HGT efficiency (Fig. 3g). This is counter-intuitive and can only be tested through systematic investigation of parameters. We have modified the manuscript based on these new results.

Reviewer #3:

Lines 96-98: It is unclear from your text whether "native comK B. subtilis PY79" is different from your xylose-inducible strain. The figure labels for figure S1 say "+ xylose / - xylose". Does this experiment represent two strains of B. subtilis (WT and xylose-inducible comK construct), or just the engineered strain with and without the inducer? If only the inducible comK strain was used for these experiments, was a qPCR or similar assay done to demonstrate a lack of leaky expression from the uninduced promoter?

We have labeled the strains clearly in the revised manuscript – engineered strain had a xylose inducible *comK* and “wild-type” strain had the same integration but with only the chloramphenicol resistance gene *cat* without *comK* (Supplementary Fig. 1a,c,d).

Lines 152-156: The filter-based experiments showing that plasmid transfer is happening by free plasmid transformation rather than conjugation are convincing. However, are there differences in plasmid DNA topology between plasmids released in vitro (likely supercoiled) and isolated plasmid DNA (likely a mix of supercoiled and non-supercoiled species). Is there a difference in transformation efficiency of supercoiled and non-supercoiled plasmids that could explain, at least in part, the noted difference in B. subtilis competence in the co-culture and monoculture treatments?

We compared the transformation efficiency of monomeric versus multimeric plasmid derived from different *E. coli* strains and found that multimeric plasmid has ~10-folds higher transformation efficiency than monomeric plasmid, which indeed explained part of the difference in the co-culture and monoculture (Fig. 1f and Supplementary Fig. 1a).

Thank you again for submitting your work to Molecular Systems Biology. We contacted the reviewers who had evaluated the initial submission (MSB-2020-10058). The previous reviewer #2 was unavailable this time, but reviewers #1 and #3 (#2 for the new submission) agreed to evaluate the revised study. We have now heard back from these two reviewers. As you will see below, they think that their previous concerns have been satisfactorily addressed. Reviewer #2 recommends some re-writing in order to present the study in a more balanced manner. We would ask you to perform these text changes in a minor revision.

On a more editorial level, we would ask you to address the following points:

Reviewer #1:

The authors have satisfactorily addressed my main concerns

Reviewer #2:

I find the authors revision satisfactory with one caveat--I would *strongly* request that the authors reframe the wording in the paper from a "microbial community" to a co-culture, especially in the title. In today's world, microbial community, even a synthetic community, implies multiple species (well-beyond two). I think the authors would agree here about this overreach. This is a liberty that the authors have taken that ultimately misleads people as to the scope and the conclusions that can be drawn from the paper. The authors changed the title since the original submission. The authors should be up front that this is a paper about co-culture in the abstract.

Reviewer #1:

The authors have satisfactorily addressed my main concerns

We thank the reviewer the valuable that helped to improve our manuscript.

Reviewer #2:

I find the authors revision satisfactory with one caveat--I would *strongly* request that the authors reframe the wording in the paper from a "microbial community" to a co-culture, especially in the title. In today's world, microbial community, even a synthetic community, implies multiple species (well-beyond two). I think the authors would agree here about this overreach. This is a liberty that the authors have taken that ultimately misleads people as to the scope and the conclusions that can be drawn from the paper. The authors changed the title since the original submission. The authors should be up front that this is a paper about co-culture in the abstract.

We thank the reviewer for the valuable comments that helped to improve our manuscript. As per the reviewer's suggestion, we changed the title to “Efficient plasmid transfer via natural competence in a microbial co-culture” and also used “co-culture” instead of “community” throughout the manuscript.

2nd Revision - Editorial Decision

9th Jan 2023

Thank you again for sending us your revised manuscript. We are now satisfied with the modifications made and I am pleased to inform you that your paper has been accepted for publication.

EMBO Press Author Checklist

Corresponding Author Name: Ophelia Venturelli
Journal Submitted to: Molecular Systems Biology
Manuscript Number: MSB-2022-11406

USEFUL LINKS FOR COMPLETING THIS FORM
The EMBO Journal - Author Guidelines
EMBO Reports - Author Guidelines
Molecular Systems Biology - Author Guidelines
EMBO Molecular Medicine - Author Guidelines

Reporting Checklist for Life Science Articles (updated January
This checklist is adapted from Materials Design Analysis Reporting (MDAR) Checklist for Authors. MDAR establishes a minimum set of requirements in transparent reporting in the life sciences (see Statement of Task: 10.31222/osf.io/9sm4x). Please follow the journal's guidelines in preparing your
Please note that a copy of this checklist will be published alongside your article.

Abridged guidelines for figures
1. Data
The data shown in figures should satisfy the following conditions:
- the data were obtained and processed according to the field's best practice and are presented to reflect the results of the experiments in an accurate and unbiased manner.
- ideally, figure panels should include only measurements that are directly comparable to each other and obtained with the same assay.
- plots include clearly labeled error bars for independent experiments and sample sizes. Unless justified, error bars should not be shown for technical
- if n<5, the individual data points from each experiment should be plotted. Any statistical test employed should be justified.
- Source Data should be included to report the data underlying figures according to the guidelines set out in the authorship guidelines on Data

2. Captions
Each figure caption should contain the following information, for each panel where they are relevant:
- a specification of the experimental system investigated (eg cell line, species name).
- the assay(s) and method(s) used to carry out the reported observations and measurements.
- an explicit mention of the biological and chemical entity(ies) that are being measured.
- an explicit mention of the biological and chemical entity(ies) that are altered/varied/perturbed in a controlled manner.
- the exact sample size (n) for each experimental group/condition, given as a number, not a range;
- a description of the sample collection allowing the reader to understand whether the samples represent technical or biological replicates (including how many animals, litters, cultures, etc.).
- a statement of how many times the experiment shown was independently replicated in the laboratory.
- definitions of statistical methods and measures:
  - common tests, such as t-test (please specify whether paired vs. unpaired), simple χ2 tests, Wilcoxon and Mann-Whitney tests, can be unambiguously identified by name only, but more complex techniques should be described in the methods section;
  - are tests one-sided or two-sided?
  - are there adjustments for multiple comparisons?
  - exact statistical test results, e.g., P values = x but not P values < x;
  - definition of 'center values' as median or average;
  - definition of error bars as s.d. or s.e.m.

Please complete ALL of the questions below.
Select "Not Applicable" only when the requested information is not relevant for your study.

Materials

Newly Created Materials	Information included in the manuscript?	In which section is the information available? (Reagents and Tools Table, Materials and Methods, Figures, Data Availability Section)
New materials and reagents need to be available; do any restrictions apply?	Not Applicable	

Antibodies	Information included in the manuscript?	In which section is the information available? (Reagents and Tools Table, Materials and Methods, Figures, Data Availability Section)
For **antibodies** provide the following information:<br>- Commercial antibodies: RRID (if possible) or supplier name, catalogue number and or/clone number<br>- Non-commercial: RRID or citation	Not Applicable	

DNA and RNA sequences	Information included in the manuscript?	In which section is the information available? (Reagents and Tools Table, Materials and Methods, Figures, Data Availability Section)
**Short novel DNA or RNA including primers, probes**: provide the sequences.	Yes	Appendix Table S3

Cell materials	Information included in the manuscript?	In which section is the information available? (Reagents and Tools Table, Materials and Methods, Figures, Data Availability Section)
**Cell lines:** Provide species information, strain. Provide accession number in repository **OR** supplier name, catalog number, clone number, and/**OR** RRID.	Not Applicable	
**Primary cultures:** Provide species, strain, sex of origin, genetic modification status.	Not Applicable	
Report if the cell lines were recently **authenticated** (e.g., by STR profiling) and tested for mycoplasma contamination.	Not Applicable	

Experimental animals	Information included in the manuscript?	In which section is the information available? (Reagents and Tools Table, Materials and Methods, Figures, Data Availability Section)
**Laboratory animals or Model organisms:** Provide species, strain, sex, age, genetic modification status. Provide accession number in repository **OR** supplier name, catalog number, clone number, **OR** RRID.	Not Applicable	
**Animal observed in or captured from the field:** Provide species, sex, and age where possible.	Not Applicable	
Please detail **housing and husbandry conditions**.	Not Applicable	

Plants and microbes	Information included in the manuscript?	In which section is the information available? (Reagents and Tools Table, Materials and Methods, Figures, Data Availability Section)
**Plants:** provide species and strain, ecotype and cultivar where relevant, unique accession number if available, and source (including location for collected wild specimens).	Not Applicable	
**Microbes:** provide species and strain, unique accession number if available, and source.	Yes	Strain information was included in "Plasmids and bacterial strains" under "Materials and Methods" section and Appendix Table S2.

Human research participants	Information included in the manuscript?	In which section is the information available? (Reagents and Tools Table, Materials and Methods, Figures, Data Availability Section)
If collected and within the bounds of privacy constraints report on age, sex and gender or ethnicity for all study participants.	Not Applicable	

Core facilities	Information included in the manuscript?	In which section is the information available? (Reagents and Tools Table, Materials and Methods, Figures, Data Availability Section)
If your work benefited from core facilities, was their service mentioned in the acknowledgments section?	Not Applicable	

Design

Study protocol	Information included in the manuscript?	In which section is the information available? (Reagents and Tools Table, Materials and Methods, Figures, Data Availability Section)
If study protocol has been **pre-registered, provide DOI in the manuscript**. For clinical trials, provide the trial registration number **OR** cite DOI.	Not Applicable	
Report the **clinical trial registration number** (at ClinicalTrials.gov or equivalent), where applicable.	Not Applicable	

Laboratory protocol	Information included in the manuscript?	In which section is the information available? (Reagents and Tools Table, Materials and Methods, Figures, Data Availability Section)
Provide DOI OR other citation details if **external detailed step-by-step protocols** are available.	Not Applicable	

Experimental study design and statistics	Information included in the manuscript?	In which section is the information available? (Reagents and Tools Table, Materials and Methods, Figures, Data Availability Section)
Include a statement about **sample size** estimate even if no statistical methods were used.	Yes	Sample size for each experiment was desribed in figure legends.
Were any steps taken to minimize the effects of subjective bias when allocating animals/samples to treatment (e.g. **randomization procedure**)? If yes, have they been described?	Not Applicable	
Include a statement about **blinding** even if no blinding was done.	Not Applicable	
Describe **inclusion/exclusion criteria** if samples or animals were excluded from the analysis. Were the criteria pre-established?<br><br>If sample or data points were omitted from analysis, report if this was due to attrition or intentional exclusion and provide justification.	Not Applicable	
For every figure, are **statistical tests** justified as appropriate? Do the data meet the assumptions of the tests (e.g., normal distribution)? Describe any methods used to assess it. Is there an estimate of variation within each group of data? Is the variance similar between the groups that are being statistically compared?	Not Applicable	

Sample definition and in-laboratory replication	Information included in the manuscript?	In which section is the information available? (Reagents and Tools Table, Materials and Methods, Figures, Data Availability Section)
In the figure legends: state number of times the experiment was **replicated** in laboratory.	Not Applicable	
In the figure legends: define whether data describe **technical or biological replicates**.	Yes	Number of biological replicates was specified for each experiment in figure legends.

Ethics

Ethics	Information included in the manuscript?	In which section is the information available? (Reagents and Tools Table, Materials and Methods, Figures, Data Availability Section)
Studies involving **human participants**: State details of **authority granting ethics approval** (IRB or equivalent committee(s), provide reference number for approval.	Not Applicable	
Studies involving **human participants**: Include a statement confirming that **informed consent** was obtained from all subjects and that the experiments conformed to the principles set out in the WMA Declaration of Helsinki and the Department of Health and Human Services Belmont Report.	Not Applicable	
Studies involving **human participants:** For publication of **patient photos**, include a statement confirming that consent to publish was obtained.	Not Applicable	
Studies involving experimental **animals**: State details of **authority granting ethics approva**l (IRB or equivalent committee(s), provide reference number for approval. Include a statement of compliance with ethical regulations.	Not Applicable	
Studies involving **specimen and field samples:** State if relevant **permits** obtained, provide details of authority approving study; if none were required, explain why.	Not Applicable	

Dual Use Research of Concern (DURC)	Information included in the manuscript?	In which section is the information available? (Reagents and Tools Table, Materials and Methods, Figures, Data Availability Section)
Could your study fall under dual use research restrictions? Please check biosecurity documents and list of **select agents and toxins** (CDC): https://www.selectagents.gov/sat/list.htm	Not Applicable	
If you used a select agent, is the security level of the lab appropriate and reported in the manuscript?	Not Applicable	
If a study is subject to dual use research of concern regulations, is the name of the **authority granting approval and reference number** for the regulatory approval provided in the manuscript?	Not Applicable	

Reporting

The MDAR framework recommends adoption of discipline-specific guidelines, established and endorsed through community initiatives. Journals have their own policy about requiring specific guidelines and recommendations to complement MDAR.

Adherence to community standards	Information included in the manuscript?	In which section is the information available? (Reagents and Tools Table, Materials and Methods, Figures, Data Availability Section)
State if relevant guidelines or checklists (e.g., **ICMJE, MIBBI, ARRIVE, PRISMA**) have been followed or provided.	Not Applicable	
For **tumor marker prognostic studies,** we recommend that you follow the **REMARK** reporting guidelines (see link list at top right). See author guidelines, under 'Reporting Guidelines'. Please confirm you have followed these guidelines.	Not Applicable	
For **phase II and III randomized controlled trials**, please refer to the **CONSORT** flow diagram (see link list at top right) and submit the CONSORT checklist (see link list at top right) with your submission. See author guidelines, under 'Reporting Guidelines'. Please confirm you have submitted this list.	Not Applicable	

Data Availability

Data availability	Information included in the manuscript?	In which section is the information available? (Reagents and Tools Table, Materials and Methods, Figures, Data Availability Section)
Have **primary datasets** been deposited according to the journal's guidelines (see 'Data Deposition' section) and the respective accession numbers provided in the Data Availability Section?	Yes	Availability of source data for primary datasets was described in the "Data availability" section.
Were **human clinical and genomic datasets** deposited in a public access-controlled repository in accordance to ethical obligations to the patients and to the applicable consent agreement?	Not Applicable	
Are **computational models** that are central and integral to a study available without restrictions in a machine-readable form? Were the relevant accession numbers or links provided?	Not Applicable	
If publicly available data were reused, provide the respective **data citations in the reference list**.	Not Applicable	
